# Radiotracers Used for the Scintigraphic Detection of Infection and Inflammation

**DOI:** 10.1155/2015/676719

**Published:** 2015-02-08

**Authors:** Chris Tsopelas

**Affiliations:** RAH Radiopharmacy, Nuclear Medicine Department, Royal Adelaide Hospital, Adelaide, SA 5000, Australia

## Abstract

Over the last forty years, a small group of commercial radiopharmaceuticals have found their way into routine medical use, for the diagnostic imaging of patients with infection or inflammation. These molecular radiotracers usually participate in the immune response to an antigen, by tagging leukocytes or other molecules/cells that are endogenous to the process. Currently there is an advancing effort by researchers in the preclinical domain to design and develop new agents for this application. This review discusses radiopharmaceuticals used in the nuclear medicine clinic today, as well as those potential radiotracers that exploit an organism's defence mechanisms to an infectious or inflammatory event.

## 1. Introduction

Since the early 1970s, the quest continues to find the ideal infection imaging and/or inflammation imaging agent. The design of such a radiotracer is usually influenced by the immunological literature, intentionally targeting the pathogen or an element of the inflammatory process, and its practical application occurs in preclinical and clinical evaluations. Some promising radiotracers have advanced beyond the clinical investigational status and achieved regulatory approval for sale to routine nuclear medicine practices. At the clinical level, the nuclear medicine role is to choose an appropriate radiopharmaceutical indicated for the patient's condition, administer the dose, and then acquire scintigraphic scans to finally derive a good medical diagnosis. An infection can be described as the combination of a pathogen invader that becomes activated and the consequent immunological response by the host. An inflammation is that process concerned with the host immune response to a pathogen and/or antigen, so as to neutralize and remove it and then initiate healing steps. The immune system is comprised of a sophisticated array of biological structures and functional strategies that serve to protect the host organism. In general, the immune system recognizes the foreign material from signals given by the pathogen (i.e., proteins, lipopolysaccharides, or microbe parts), signals emitted from the damaged site (i.e., increased local blood flow, release of eicosanoids, cytokines, etc., from local cells), or other immune elements (i.e., complement binding to antibodies attached to microbes, opsonization, chemotactic molecules, etc.) to circulating and localized “sentinel” cells.

**Figure 1:**
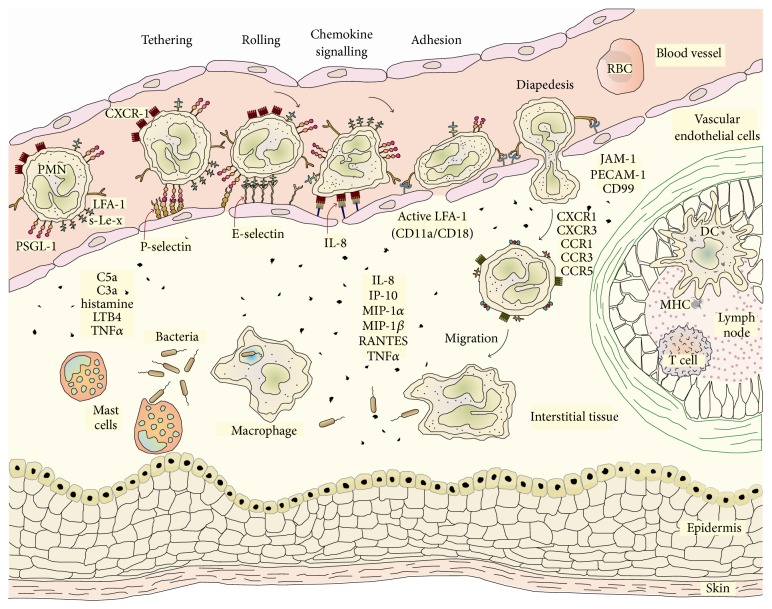
Schematic representation of an immune response to a bacterial infection. Tethering: P-selectins bind to PSGL-1 and tether PMN. Rolling: P-selectins bring PMN near E-selectins and slow rolling commences. Chemokine signalling: slow rolling allows IL-8 binding to CXCR1, then integrin activation results in more adhesive interactions with endothelial ligands. Adhesion: activated LFA-1 binds ICAM-1 or ICAM -2 for firm adhesion. Diapedesis: PMN begins diapedesis by exchanging tight junction molecules with endothelial cells. Migration: PMN follows a gradient of inflammatory chemokines to pathogens. Attack: complement attacks the incoming pathogens, mast cells, and macrophage phagocytose pathogens; toll-like receptor molecules trigger inflammation; antigen presenting dendritic cells express MHC molecules to activate T lymphocytes and to recruit neutrophils. PMN = polymorphonuclear neutrophil; CXCR/CCR = chemokine receptor; s-Le-x = sialyl-Lewis X; LFA = lymphocyte function-associated antigen; PSGL = P-selectin glycoprotein ligand; IL = interleukin; CD = cluster of differentiation; CD11a/CD18 = integrins; RBC = red blood cell; JAM = junctional adhesion molecule; PECAM = platelet endothelial cell adhesion molecule; DC = dendritic cell; MHC = major histocompatibility complex; T cell = T lymphocyte; IP-10 = interferon-gamma induced protein-10; MIP = macrophage inflammatory protein; RANTES = regulated on activation, normal T cell expressed and secreted (CCL5 chemokine); TNF*α* = tumor necrosis factor alpha; C3a and C5a = complement components 3a and 5a; LTB4 = leukotriene B4.

The acute phase response occurs during the recognition period when the host immune system commences complex attack strategies on the antigenic material (i.e., phagocytosis, killer lymphocyte actions, etc.). Antigens can be xenobiotic molecules, small or large allogeneic molecules, error-assembled autologous molecules, small or large particulates, or even macrosized materials. Pathogens can be microbes (i.e., bacteria, viruses, prions, viroids) or larger multicellular organisms (i.e., fungi, nematodes, other parasites). The defence mechanism toward a sudden presence of foreign material involves the appearance of naïve dendritic cells that subsequently internalize some extracellular antigen to create major histocompatibility complex (MHC) molecules. These tightly bound complexes are expressed on the outer membrane surface, and this is associated with the differentiation of naïve dendritic cells into a mature state in the lymph nodes. Here, dendritic cells communicate their recognition of antigen with other linked cells (i.e., lymphocytes), using proinflammatory cytokines (i.e., tumour necrosis factor-*α*, interferon-*γ*, interleukins, etc.) to ultimately recruit neutrophils from the circulation to the invasion site (Figure 1). Mast cells at the site are stimulated into releasing histamines that cause vasodilation. At the endothelial cell barrier, neutrophils participate in rolling, adhesion, and transmigration events into the extravascular site that harbours the antigen. The chronic phase of the immune response follows with a cascade of simultaneous attack events involving both adaptive and innate systems. The leucocyte* milieu* is actively engaged in a complex sequence of intense cell-cell signalling to recruit polymorphonuclear cells, as well as numerous direct and indirect actions to control, degrade, and remove the antigen. Among those, macrophages and T helper cells are stimulated into releasing inflammatory mediators, destructive enzymes (i.e., myeloperoxidase, xanthine oxidase, etc.), and oxidizers (i.e., superoxide anions, nitric oxide, hydrogen peroxide, N-chloroanalogues). The activated complement system participates via an increase in immunoglobulin production and chemotactic mediators to recruit more neutrophils. After the antigen is removed, the healing phase initiates with mast cells, T-helper cells, and basophils that release interleukin signals, then stimulated macrophages express tissue remodelling molecules and angiogenic growth factors. There is a promotion of fibroblast cells growth, these cells accumulate at the wound site and proliferate into myofibroblasts followed by collagen deposition. T lymphocytes and monocytes are recruited to the site, macrophages alternate their responses and angiogenesis is promoted during repair.

Immunology is a major research field, widely pursued to expand our understanding of cellular and molecular processes in immunity, which can be translated into improving human health in medical diagnoses and treatments. The advancing knowledge is correlated with an impetus to develop radiotracers as inflammatory response markers; some have already progressed to the clinic as radiopharmaceutical-white cells, immunoglobulins, complement proteins, cytokines, or microbial peptide fragments. A radiopharmaceutical is an agent that has achieved the market authorization for human use and is comprised of a medical radionuclide bonded to a pharmaceutical ligand. The medical radionuclide typically emits safe *γ*-rays with a minimal radiation burden, and it is appropriately detected by a scintillation gamma camera for processing data into patient scans. Regulatory-approved diagnostic radiopharmaceuticals such as ^67^Ga-citrate and ^111^In-oxine have been commercially available in various continents since the ~1970s, following a time of newly formed regulatory agencies and during the “revolution” of others. In the 1980s CERETEC was a new product release, HIG, LLK, Scintimun, and Sulesomab followed during the 1990s, and then in 2004 ^18^FDG was introduced to clinics in an era that coincided with regulatory efforts to achieve global GMP harmonisation. Radiopharmaceuticals can contain active ingredients of synthetic origin, biological origin, or a formulation to label leukocytes, and are traditionally produced in a Good Manufacturing Practice (GMP) facility. These injectable products exploit the inflammatory response by radiolabeling leukocytes, participating in increased metabolic pathways, and labeling endogenous molecules or microbes. Information about a typical radiopharmaceutical product is accessible from the Product Information Sheet or company website, and the academic literature contains abundant information on their clinical applications.

Clinical investigational radiopharmaceuticals, or tracers late in the development phase, are intended for human use and usually have local human ethics approval or in some countries low-level regulatory approval, but nevertheless they do not achieve the market authorization. Preclinical radiotracers are radiochemicals, or tracers in the research phase; they are intended for animal use and have local animal ethics approval, or for* in vitro* bench work. The growing literature contains information on radiochemicals such as synthetic molecules, biologicals, and cells, ranging from their radiolabeling methodologies to localisation mechanisms* in vivo*. In this chapter the routine clinical products are discussed, plus those radiotracers capable of effectively discriminating infection from inflammation or selectively discriminating between different types of infection.

## 2. Radiopharmaceuticals

### 2.1. Current Clinical Imaging Agents in Routine Use

#### 2.1.1. Synthetic Molecules

The most commonly used diagnostic radiopharmaceuticals are mainly ^99m^Tc-based. The tracer is either used to radiolabel blood cells* ex vivo* before intravenous injection into the patient, or is administered directly. Technetium-99m is obtained from a generator, a reuseable device that gives the parent isotope as sodium ^99m^Tc-pertechnetate pharmaceutical, from the grandparent ^99^Mo-molybdate (Table 1). Its mode of decay is by isomeric transition to emit *γ*-rays of 140 keV with no tissue burden. This point source can be easily shielded (i.e., 7 mm Pb pots) to protect operators, and the isotope half-life of 6 hours is convenient during a clinical working day. ^99m^Tc-pertechnetate requires reduction* in situ* to allow radiometal coordination with a ligand. Another important isotope Indium-111 is supplied as a salt solution, or in the form of a coordinated complex such as ^111^In-oxine to radiolabel leukocytes. This isotope decays by electron capture to yield useful energies of detection with a *γ*-camera, and it needs more shielding than ^99m^Tc during handling by the operator. The 3-day half-life has the advantage of performing delayed patient scans after significant accumulation of the radiopharmaceutical. Gallium-67 is predominantly supplied as the citrate complex in a multidose vial, the isotope decays by electron capture to yield detectable energies, and it requires thicker shielding than ^99m^Tc to contain the higher energy emissions. Like ^111^In, the half-life of ^67^Ga also allows for delayed scintigraphy. The currently popular isotope ^18^F decays by emitting positrons that collide with surrounding atoms and annihilate to produce higher energy *γ*-rays (2 × 511 keV). It requires considerably more lead shielding (i.e., >20 mm Pb), and the half-life of 110 min necessitates swift coordination of operations during a working day.

Of the commercial cold kit formulations (Table 2), CERETEC is used to prepare ^99m^Tc-exametazime (also known as ^99m^Tc-HMPAO) indicated for perfusion brain imaging, but extensive validation has also led to its widespread application of radiolabeling white blood cells to detect infection. The vial formulation contains the active ingredient exametazime, with excipients stannous chloride and sodium chloride under a nitrogen gas atmosphere. The active is present as the d,l-diastereoisomers, the ligands that coordinate ^99m^Tc-reduced by stannous ions. The reconstituted product is tested soon after reconstitution. Quality control assays have employed a paper stationary phase with a single strip [1] developed in an organic solvent, a cartridge method with saline solvent [2], or the manufacturer recommended dual strip method [3] with methyl ethyl ketone and normal saline solvents. The traditional formulation is used within thirty minutes after reconstitution to label white blood cells in an* ex vivo* procedure.

LLK is a product used to prepare ^99m^Tc-stannous fluoride colloid, a reagent for preparing ^99m^Tc-leukocytes indicated for inflammation/infection imaging. There are two component vials in the kit: the vial A formulation contains aqueous sodium fluoride solution and vial B contains lyophilized stannous fluoride. The ^99m^Tc-colloid shelf-life is 60 minutes at room temperature, and it is quality control tested using a silica gel impregnated-paper strip with saline solvent [4] to give >95% radiochemical purity (RCP). Soon after preparation it is used for labeling white cells.

MDP is reconstituted with ^99m^Tc-pertechnetate to yield ^99m^Tc-medronate, a product indicated for bone imaging. The bone scan provides information for comparison with the primary scan derived from another agent (i.e., ^67^Ga-citrate). A typical vial formulation contains medronic acid (MDP), stannous chloride, and ascorbic acid under nitrogen. MDP is likely to be mixtures of short- and long-chain polymers [5]. ^99m^Tc-MDP tends to degrade by oxidation and radiolysis unless ascorbic acid is part of the formulation. The reconstituted shelf-life is 8 hours at room temperature, and quality control testing using a dual strip method gives an RCP of ≥95% ^99m^Tc-MDP and ≤2% of unreacted ^99m^Tc-pertechnetate impurity.

^111^In-oxine is a radioactive solution indicated for white cell imaging/counting after preparing ^111^In-leukocytes. The formulation comprises 8-hydroxyquinoline, polysorbate 80, and HEPES buffer in 0.75% sodium chloride solution under nitrogen. Oxine is a bidentate ligand that forms a tris-complex with ^111^In at neutral pH (~7). The polysorbate excipient solubilizes the hydrophobic moiety in aqueous solution. The shelf-life is 8 hours at 2–8°C after opening, and the radiopharmaceutical is calibrated at 37 MBq/mL (>1.85 GBq/*μ*g) with an impurity level of ≤0.08%  ^114m^In per ^111^In. The product is often used soon after receipt to radiolabel the leukocytes, but it is not approved for direct administration into a patient.

^67^Ga-citrate is also a radioactive solution, indicated for detecting cancer and some acute inflammatory lesions after patient injection. The radioactive formulation of sodium chloride and sodium citrate is isotonic and usually contains an antimicrobial preservative such as benzyl alcohol (0.9% w/v) with a broad pH range (4.5–8). ^67^Ga-citrate is essentially a bidentate complex at neutral pH; the citrate salt is designed to minimize the level of gallate (^67^Ga(OH)_4_^−^) and gallium hydroxide (^67^Ga(OH)_3_). The product is available as different calibrated amounts, each multidose vial should be used within 5-6 days. Paper electrophoresis [6] is one quality control test method; otherwise a single strip of Whatman 1 paper developed in pyridine : ethanol : water [1 : 2 : 4] solvent can determine a radionuclidic purity ≥99.8%.

^18^F-fluorodeoxyglucose (^18^FDG) is a radioactive solution indicated for the identification of regions of abnormal glucose metabolism associated with the myocardium, foci of epileptic seizures, and malignancy. A typical ^18^FDG injection is an isotonic, sterile, pyrogen-free, and preservative-free solution, containing the active ingredient 2-[^18^F]fluoro-2-deoxy-D-glucose in a broad pH range (4.5–8). This product is stored below ~25°C and used on the same day within 10–12 hours due to the short half-life of ^18^F (*t* = 110 minutes). ^18^FDG is calibrated at different GBq levels; the manufactured amount is primarily dictated by the advanced number of patient bookings. Quality control testing is performed by the manufacturer or at the end-user site, and it involves identification tests (i.e., radionuclidic purity, half-life, radiochemical purity), pH, assays (i.e., residual solvents, impurities), and biological tests (i.e., sterility, endotoxins).

#### 2.1.2. White Blood Cells

Different countries vary in their regulations, laws, or acts regarding extemporaneous compounding of radiopharmaceuticals; however, there are special requirements when radiolabeling autologous blood cells because sterilization is not possible after the process. Biological fluids are handled aseptically in a biohazard safety cabinet containing a work zone supplied with HEPA-filtered air (vertical laminar flow), separate from the area for compounding synthetic radiopharmaceuticals. On the day of a scan, the patient donates a whole blood sample and then it is manipulated to isolate the white cells, in the first part of a two-part procedure. The blood syringes have a wide bore needle (<21 G) attached, to prevent cellular damage from shear stress during fluid transfers. Only trained operators perform the procedure under controlled conditions, to prevent cross contamination to the operator from a potentially infectious sample, and the blood sample from the working environment or operator. The second part of the procedure involves labeling the isolated white cells with a selected radiopharmaceutical. Usually one operator is assigned per cell labeling procedure that is performed in a dedicated and clean work zone. There should be written procedures and regular practice to ensure that the original blood donor (patient) receives their own tagged cells. A radiolabeled-leukocyte patient dose must be a homogeneous suspension and absent of any clumps or cell aggregates, and it should be injected into the donor patient as soon as possible after the procedure, certainly within 1 hour so that there is minimal release of radioisotope from cells* in vitro*. 

*(1) Radiopharmaceuticals for Labeling White Cells (Ex Vivo Procedures)*

^99*m*^ *Tc-HMPAO*. ^99m^Tc-HMPAO is a common radiopharmaceutical that has largely replaced ^111^In-oxine because of the favourable physical properties of ^99m^Tc, wide availability, and cost and lower radiation burden to the patient. Its lipophilic property allows it to cross the white cell membrane, where the isotope is intracellularly trapped. The labile ^99m^Tc-HMPAO agent must be rapidly combined with white cells to initiate radiolabeling, when the hydrophilic impurities are low. The impurities are physically separated from ^99m^Tc-cells during the purification step. Quality control testing is performed by the operator prior to release and involves an identification test (i.e., visual inspection) and assay (i.e., radiolabeling efficiency or %RE). 

^99*m*^ *Tc-Tin Fluoride Colloid*. ^99m^Tc-tin fluoride colloid originated as a radiotracer for labeling leukocytes in Australia almost 30 years ago and has since been employed in routine clinical practice. The reagent ^99m^Tc-colloid is prepared in advance to yield large radiocolloid particles that have an affinity for cells. Unlike ^99m^Tc-HMPAO and ^111^In-oxine that are added to the isolated mixed leukocytes, ^99m^Tc-tin fluoride colloid is added directly to whole blood. This radiocolloid binds to the cell membrane, and then it is potentially phagocytosed by the polymorphonuclear cells. Any unbound radioactivity (<5%) is removed during a purification step. Quality control testing involves visual inspection and determining %RE. 

^*111*^ *In-Oxine*. ^111^In-oxine is neutral and lipophilic, allowing it to diffuse through the white cell membrane, then ^111^In(III) is released and ~77% [7] trapped after binding with cytoplasmic molecules. Labeling of mixed leukocytes causes some radiation damage to the lymphocyte population due to self-irradiation by internal conversion of (Auger) electrons, however, damaged cells are eliminated through apoptotic and phagocytic pathways [8]. As in all cell radiolabeling procedures, the purification step physically separates cells from soluble impurities. Quality control testing traditionally involves visual inspection and determining %RE. 

*(2) Radiopharmaceutical for Labeling Other Cells In Vivo*. ^67^Ga-citrate is administered directly into the circulation by intravenous injection, and the isotope is transchelated to more avid serum proteins, especially transferrin. It has been proposed that ^67^Ga-transferrin delivers the radionuclide to circulating leukocytes and then transchelation to intracellular lactoferrin affords labeled white cells. Furthermore ^67^Ga-transferrin most likely provides ^67^Ga to ferritin or siderophore molecules expressed by bacteria at the lesion site. ^67^Ga has higher chemical affinity for siderophores > ferritin > lactoferrin > transferrin, and this explains transchelation events between such molecules* in vivo*.

#### 2.1.3. Molecules of Biological Origin

*LeukoScan.* LeukoScan is a product used to prepare ^99m^Tc-sulesomab, indicated for imaging infection/inflammation in the bone of patients with suspected osteomyelitis, including those with diabetic foot ulcers. A typical vial formulation contains the active ingredient sulesomab (IMMU-MN3), stannous chloride, tartrate and acetate salts, sodium chloride, glacial acetic acid and hydrochloric acid (trace) and sucrose under nitrogen. IMMU-MN3 is a murine Fab'-SH antigranulocyte monoclonal antibody fragment. The reconstituted shelf-life is 4 hours at room temperature and quality control testing can be performed using a single strip method in acetone solvent to give ≥90% RCP ^99m^Tc-sulesomab (and <10% free ^99m^Tc). ^99m^Tc-sulesomab binds to the nonspecific cross-reacting antigen (NCA) 90 surface glycoprotein of activated granulocytes and then ^99m^Tc-cells participate in the immune response. This product also recognizes endogenous carcinoembryonic antigen (CEA). After intravenous injection, radioactivity accumulates at the inflammatory site over the next 1–8 hours. Possible contraindications are a lowered white blood cell count, and a human-anti-mouse antibody response (HAMA) since the antibody fragment is a xenobiotic.

*Scintimun.* Scintimun is a product used to prepare ^99m^Tc-besilesomab that is indicated for imaging the location of infection/inflammation in peripheral bone of patients with suspected osteomyelitis. There are two component vials in the Kit and each vial contains a white powder. The* Scintimun* vial formulation contains besilesomab (BW 250/183), phosphate salts, and sorbitol under nitrogen. BW 250/183 is a murine immunoglobulin of the IgG1 isotype, a monoclonal antibody. The* Solvent for Scintimun* vial formulation contains 1,1,3,3-propane tetraphosphonate salt, stannous chloride, and sodium chloride under nitrogen. The reconstituted shelf-life is 3 hours at room temperature, and the quality control test of the injection occurs with a single strip developed in MEK solvent to give ≥95% RCP ^99m^Tc-besilesomab (and <5% free ^99m^Tc). ^99m^Tc-sulesomab in the blood circulation binds to the NCA-95 on granulocytes and granulocyte precursors, and also recognizes CEA-related cell adhesion molecule-8. Radiolabeled cells* in vivo* participate in the immune response and accumulate at the inflammatory site over the next 3–6 hours. As with LeukoScan, possible undesirable effects are a HAMA response, anaphylactic or anaphylactoid reactions. 

*Nanocoll*. Most commercial organic particulates are comprised of the denatured or aggregated protein human serum albumin. The general procedure for larger particles such as human albumin microspheres requires rapid mixing of an aqueous albumin solution with vegetable oil to produce an emulsion of spheres. In the presence of stannous chloride the oil is heated to remove water; then spheres solidify with the reductant enmeshed within the colloidal matrix [9]. The spheres are filtered, washed with a nonpolar solvent to remove excess oil, and then sieved to isolate the appropriate size range. Nanocoll is a product used to prepare ^99m^Tc-albumin nanocolloid injection, indicated for inflammation scanning in areas other than the abdomen. A typical vial formulation contains human albumin colloid, stannous chloride, poloxamer 238, sodium phosphate dibasic, sodium phytate, and glucose under nitrogen. The reconstituted shelf-life is 6 hours at room temperature, and quality control testing can be performed using a single strip (Whatman 1 paper) developed in methanol : water [85 : 15] solvent to give ≥95% RCP of product. ^99m^Tc-albumin nanocolloid after intravenous injection is delivered by blood flow at or near the inflammatory site due to the “leaky” vasculature, 45 to 60 minutes later. Possible undesirable effects are hypersensitivity to the active substance or to any of the excipients. Human albumin in Nanocoll is nonreactive towards hepatitis B surface antigen, and antibodies towards the human immunodeficiency virus and hepatitis C virus.

### 2.2. Radiolabeling Methodologies

#### 2.2.1. Simple Operations

A simple operation involves those functions undertaken by personnel during extemporaneous compounding of either one radioisotope reconstitution/radiolabeling step and then withdrawal of final patient dose(s) from the source vial, or no radiolabeling step but withdrawal of doses directly from the source vial. Examples are ^99m^Tc-HMPAO, ^99m^Tc-MDP, ^99m^Tc-sulesomab and ^99m^Tc-albumin nanocolloid. Reconstitution of CERETEC with freshly eluted ^99m^Tc-pertechnetate (<2 hours old) yields >90% d,l-diastereoisomeric mixture of ^99m^Tc-HMPAO within the first few minutes, and ≥80% during the 30-minute shelf-life. ^99m^Tc(VII) pertechnetate is reduced by stannous ions and the resulting radiometal-bound-HMPAO contains a mono-oxo core of ^99m^Tc(V)=O. ^99m^Tc-MDP is prepared efficiently after reconstitution with concentrated ^99m^Tc-pertechnetate. The resulting complex is a polymeric structure [5] with subunits of [^99m^Tc(OH)MDP^−^]_*n*_. The presence of an antioxidant (i.e., ascorbic acid) in the formulation solution acts to scavenge any free radicals formed by oxidative radiolysis, and this enhances the product stability at room temperature over its shelf-life. ^99m^Tc-sulesomab is prepared in good yield at 10 minutes after reconstitution with concentrated ^99m^Tc-pertechnetate. The ^99m^Tc oxidation state and the type of bond to antibody fragment are unknown, but the reduced radiometal is likely to be coordinated with the sulfurhydryl groups in the Fab' fragment (see Figure 4). ^99m^Tc-albumin nanocolloid is prepared in high yield, the stannous ions within the particle matrix effect reduction before ^99m^Tc coordinates with ionic groups on the particle surface. The presence of phytate in the formulation suggests this weak coordinator probably binds reduced ^99m^Tc transiently, in advance of binding to the surface of particles that are essentially <80 nm in size. ^111^In-oxine, ^67^Ga-citrate, and ^18^FDG (off-site cyclotron) are produced and quality control tested at a GMP manufacturing site, allowing the released product to be used immediately after delivery at the imaging clinic.

#### 2.2.2. Complex Operations

A complex operation involves those functions undertaken by personnel during extemporaneous compounding with multiple steps to prepare radiopharmaceutical patient doses. This particularly includes blood cells that require handling, manipulation, isolation, radiolabeling, and purification steps before withdrawing a patient dose, and these occur in a higher grade work environment (laminar flow cabinet, biohazard cabinet, isolator, cleanroom). Examples include ^99m^Tc-leukocytes and ^111^In-leukocytes. The noncellular tracers also need multiple steps for their preparation, such as ^99m^Tc-tin fluoride colloid and ^99m^Tc-besilesomab. When a facility has a cyclotron on-site it can produce ^18^F-fluoride, the starting material for synthesizing ^18^FDG by an automated system, but it is nevertheless a complex process that includes heating and purification of materials. 

*LLK &* ^99*m*^ *Tc-Tin Fluoride Colloid-Leukocytes.* Two radiolabeling procedures are performed when leukocytes are bound with ^99m^Tc-tin fluoride colloid. The first involves mixing aqueous sodium fluoride with a solution of stannous fluoride in water in the Kit B vial. Then the diluted contents of the vial are passed through a 0.2 *μ*m filter, and a portion of the filtrate is added to ^99m^Tc-pertechnetate. After mixing the liquid by rotation in a syringe for 50 minutes, ^99m^Tc-pertechnetate is entirely converted to the colourless and hydrophilic ^99m^Tc-tin fluoride colloid. The chemical reactions of particle formation are characterized by two sequential processes of nucleation and growth [10]. In a supersaturated concentration of stannous fluoride solution, molecules associate in clusters or embryos until they reach a size threshold when they precipitate as “template” particles [11]. After nucleation, the particles grow from the diffusion of remaining reactants to template surfaces. Tin-oxide bonds in tin clusters have been identified in the Kit B formulation by mass spectrometry, and these bonds are important in the particle structure [12]. The filtration step in the procedure is important in concentrating <200 nm particles intended for reaction with ^99m^Tc-pertechnetate. In contrast to other preformed particles such as albumin nanocolloid, ^99m^Tc-tin fluoride colloid particles are created simultaneously when ^99m^Tc is reduced, and then radiometal binds to the oxygenated particle surface. Stannous fluoride is hydrolysed rather than oxidized, the ensuing Sn–O–Sn and Sn(II)–OH bonds occur during radiocolloid formation and growth. Sodium fluoride in vial A provides sodium ions for the surface containing Sn–O^−^ bonds. Radioactive particle size analyses indicate ≥95% of ^99m^Tc-particles are 1000–3000 nm [12], and it is still unclear whether the growth phase is defined as an association of many 200 nm template particles to produce one larger 2000 nm particle, or that each template particle enlarges to a diameter of 2000 nm. This radiocolloid is particularly interesting because depending on its physical manipulation, it either deflocculates into smaller particles [13] or aggregates into larger particles [14]. These observations indicate that certain bonds in the colloid are weak and perhaps even reversible, implying that particle growth involves the association of many template particles.

In the second part of the procedure, ^99m^Tc-tin fluoride colloid is mixed for an hour with a sample of patient whole blood* ex vivo*, cells are radiolabeled, the sample is centrifuged to sediment the cells away from free ^99m^Tc, and then reconstituted blood is administered back into the same patient. After using two different techniques [15, 16], it was shown that ^99m^Tc-activity is predominantly associated with red cells (66–75%), to a lesser extent neutrophils (9–14%), and other white cells (10–12%). It was reported that the neutrophils could scavenge radiocolloid bound with lower affinity to the erythrocyte surface. However, the radiolabeling mechanism was proposed to depend upon phagocytic engulfment [17] of appropriately sized ^99m^Tc-tin fluoride colloid particles by neutrophils, monocytes, and eosinophils. Another work has shown that the opsonization of ^99m^Tc-tin fluoride colloid is an important process in blood prior to phagocytosis and that the opsonin concentration in the small blood sample* ex vivo* might be insufficient to account for the total population of antigen particles [18]. Other attempts to support this hypothesis showed the proportion of radioactive neutrophils in whole blood was unchanged, regardless of the presence or absence of the phagocytic inhibitor cytochalasin B. Furthermore, a preliminary report showed ^99m^Tc-labeled-neutrophils had an increased response to LPS [19], a bacterial lipopolysaccharide that activates biochemical reactions involved with phagocytosis. Whether phagocytosis completely explains the cell radiolabeling mechanism is debatable, because the percentage of ^99m^Tc-neutrophils in whole blood* in vitro* is similar when the incubation is performed either at room temperature [15] or 4°C [16] via different techniques. At the lower temperature particle internalization is inhibited, although cell surface attachment can occur [20–22]. Specific cell surface adherence is the favoured mechanism by some researchers [23]. The evidence in the literature when considered as a whole does, however, indicate that ^99m^Tc-tin fluoride colloid is surface bound to red and white cells in blood [15] and especially for the polymorphonuclear cells, this is a priority before any internalization.

*Scintimun.* ^99m^Tc-besilesomab can be prepared in high yield soon after reconstitution in a three-step process. The contents in the* Solvent for Scintimun* vial are dissolved when reconstituted in saline, and one-fifth of this reductant formulation is subsequently transferred into the* Scintimun* vial containing besilesomab powder. ^99m^Tc-pertechnetate is added to the dissolved besilesomab, the radiometal is reduced and then binds to the antibody. In the* Scintimun* vial besilesomab is present in a partially reduced form, with free sulfurhydryl groups in the hinge region that coordinate reduced ^99m^Tc. The ^99m^Tc oxidation state and the type of bond to antibody are unknown, but metal coordination may also involve oxygen and nitrogen atoms oriented favourably in the structure. 

^99*m*^ *Tc-HMPAO-Leukocytes*. ^99m^Tc-HMPAO must be used within its 30-minute shelf-life, otherwise its gradual decomposition results in high levels of ^99m^Tc-pertechnetate and a hydrophilic ^99m^Tc-secondary complex. These impurities are not useful for labeling leukocytes. This method is different to the ^99m^Tc-tin fluoride colloid-leukocytes procedure because white blood cells are isolated in advance of their incubation with ^99m^Tc-HMPAO. This is achieved by adding a high molecular weight plasma volume expander (i.e., 2-hydroxyethyl starch, dextran 70, etc.) to a larger sample of anticoagulated whole blood and then the erythrocytes sediment over the next 30–45 minutes. The supernatant leukocyte rich plasma is removed from the erythrocytes layer and then centrifuged at low speed to sediment the white cell pellet. The pellet is isolated from the supernatant containing the sedimenting agent [24], resuspended in a small volume of saline, and then concentrated ^99m^Tc-HMPAO solution is mixed into the leukocyte suspension. Ten minutes of incubation at room temperature allows the radiotracer to bind with leukocytes, by diffusing through the amphipathic phospholipid membrane and then becoming trapped inside the cell. Retention of ^99m^Tc within a white cell is likely from either a glutathione-reduction of ^99m^Tc-HMPAO into the hydrophilic ^99m^Tc-secondary complex, and/or ^99m^Tc-HMPAO-binding to organelles/nondiffusible cytoplasmic proteins. Approximately 70–80% of the radioactivity is bound to granulocytes. If a higher ^99m^Tc-granulocyte population is required, then a gradient solution can be used during the centrifugation step to separate and enrich the granulocyte fraction. 

^*111*^ *In-Oxine-Leukocytes.* ^111^In-oxine labeling succeeds when leukocytes are separated from whole blood containing transferrin. Similar to the ^99m^Tc-HMPAO procedure above, the mixed leukocytes (or enriched granulocytes) pellet is resuspended in a small volume of saline and then buffered ^111^In-oxine is added directly into the suspension. Ten minutes of incubation at room temperature allows the radiotracer to cross the white cell membrane, ^111^In binds to the more avid-chelating cytoplasmic components (i.e., lactoferrin), and eventually free 8-hydroxyquinoline is released from the cell. Any unbound ^111^In-oxine is removed by centrifugation, and the resuspended pellet becomes the patient dose in an expected yield of 50–80%.

^*18*^ *FDG.* A cyclotron produces* carrier-free* ^18^F-fluoride (^18^F^−^) ions in water. Nucleophilic substitution is the chemical reaction that occurs between ^18^F^−^ and an analogue of 2-OH-mannose. From the cyclotron, aqueous ^18^F^−^ solution is pneumatically delivered inside the clean work zone of a hot cell, into a cassette module where the process reactions occur. Firstly, ^18^F^−^ is isolated from the aqueous solvent by trapping it on an ion-exchange column, and then it is eluted with acetonitrile solvent containing 2,2,2-cryptand and potassium carbonate. Solvent evaporation of the eluate gives a very reactive radiofluorination agent [(crypt-222)K]^+^. ^18^F^−^, the heterocyclic cyrtand has a strong affinity for potassium ions and results in more exposed fluoride anion electrons. The other reagent loaded into the cassette is the 2-triflated mannose; it contains the appropriate leaving group (triflyl rather than hydroxyl) for nucleophilic substitution with ^18^F^−^. After heating the triflated mannose with [(crypt-222)K]^+^. ^18^F^−^ in acetonitrile at ~85°C for 5 minutes, an intermediate tetra-O-acetylated-2-^18^FDG is yielded. The intermediate is loaded onto a C_18_ cartridge, and then purified from unreacted ^18^F^−^ and cryptand impurities that are eluted off with solvents flowing to waste. The next eluent of hydroxide solution initiates an alkaline hydrolysis reaction to remove four acetyl groups, and the last elution with water extracts the crude glucose product. After adjusting the pH and osmolality of the eluate, purification of the formulation occurs through a second C_18_ cartridge and then on an alumina-N cartridge before aseptic filtration (0.2 *μ*m filter) into the final product container. The automated process to radiosynthesize ^18^FDG from ^18^F^−^ takes approximately 30–40 minutes. Some facilities autoclave the dispensed ^18^FDG product to avoid sterility testing each batch. Quality control testing occurs over the next ~2 hours after production, and before interim release for human use.

#### 2.2.3. Advantages and Disadvantages of Methodologies

^67^Ga-citrate and ^18^FDG (supplied) can be used to withdraw patient doses from the stock vial on the day of receipt (Table 3). No blood cells are involved and therefore no bioburden is endured from potentially contaminated samples, although thicker lead shielding for minimizing the radiation burden makes it cumbersome for operators to handle the heavier items. ^111^In-oxine is not administered to patients directly but is the necessary reagent to prepare ^111^In-leukocytes. The lengthy procedure involves many steps where a needle stick injury could occur; the operator should be vigilant of the sample that might contain infectious pathogens. Another disadvantage using ^111^In is that more shielding is required to minimize the radiation burden. ^99m^Tc-MDP is rapidly prepared after reconstitution, and it can be promptly administered to patients. In a regular radiopharmacy that prepare other ^99m^Tc-products, no special equipment is required for handling or minimising the radiation burden from ^99m^Tc-MDP, and no blood cells occlude any special precautions by the operator.

^99m^Tc-HMPAO is prepared quickly, whereas the following cell labeling procedure takes ~2 hours. There is low radiation burden but a possible bioburden to prepare ^99m^Tc-HMPAO-leukocytes. ^99m^Tc-tin fluoride colloid preparation takes one hour and then leukocyte labeling adds another hour before the patient dose is ready, some special equipment is also required. ^99m^Tc-sulesomab, ^99m^Tc-besilesomab, and ^99m^Tc-albumin nanocolloid are easily prepared with no radiation burden, neither require blood cells as they are injected directly into the bloodstream, and therefore no bioburden is incurred by the operator. With formulations that contain allogeneic biological material, there is a possibility that a patient endures undesirable effects (i.e., bioburden such as HAMA, hypersensitivity, etc.), and for some clinics, synthetic tracers are preferred with or without autologous leukocytes. The lengthiest preparation of ~2.5 hours concerns ^18^FDG when the cyclotron is located on-site, thicker shielding minimizes a high radiation burden, and complex, expensive equipment or consumable items are necessary within a supporting infrastructure.

### 2.3. *In Vivo* Distribution & Uptake Mechanisms

#### 2.3.1. Clinical Radiotracers

^99*m*^ *Tc-MDP*. Three hours after intravenous administration this ^99m^Tc-phosphonate is essentially located in the blood with 1–3% bound to plasma proteins, ≤59% is excreted in urine, and ~45% remains in the skeleton with a high bone-to-soft tissue ratio. The polymeric anionic structure [^99m^Tc(OH)MDP^−^]_*n*_ governs the mechanism of uptake by binding to Ca(II) ions in calcium hydroxyapatite crystals comprising bone. Also, localisation occurs in the mineral phase of bone with low binding to the organic matrix. The integrity of radioactive bone uptake is altered in the region of interest when an infection or inflammation is present.

^99*m*^ *Tc-Sulesomab.* At one hour after infusion of ^99m^Tc-sulesomab there is 34% of radioactivity in the blood, it decreases to 17% at 4 hours and further to 7% at 24 hours. ^99m^Tc-sulesomab recognizes an antigenic structure on the surface of granulocytes, common to the glycoprotein NCA-90 and CEA. There is a higher affinity of the radiotracer for activated rather than resting granulocytes; the radiolabeling reaction* in vivo* may occur at the local inflammatory site rather than to circulating granulocytes [25]. The ^99m^Tc-sulesomab-labeled-granulocytes have the same biological activity as unlabeled-granulocytes, and these participate in the immune response at the osteomyelitic site. There is an accumulation of ^99m^Tc-sulesomab-granulocytes at the inflammation over the next one-to-eight hours as the tracer leaves the blood circulation. Nearly half (41%) of the injected radioactivity is detected in the urine at 24 hours, indicating the ^99m^Tc-sulesomab-granulocytes are eventually metabolized and renally excreted. 

^99*m*^ *Tc-Albumin Nanocolloid.* After intravenous injection of radiocolloid particles into the bloodstream, they readily interact with complement proteins to become opsonized and provide a recognition signal for localized phagocytes. There is essentially quantitative uptake by the reticuloendothelial tissues such as the liver, spleen, and bone marrow, with little or no urine excretion after 30 minutes. The reticuloendothelial or* mononuclear phagocyte* system consists of primarily monocytes and macrophages that engulf radiocolloid upon presentation. The relative distribution between organs depends on radiocolloid size, where larger particles in the size range are retained by the liver and finer particles are enriched in bone marrow. In terms of an immune response to an inflammatory event, ^99m^Tc-albumin nanocolloid is delivered from the circulation to the endothelial cell barrier near the extravascular inflammation site; the tissue phagocytes concentrated at the site are initiated to move toward the barrier where the newcomer radioantigen is presumably engulfed as part of the retention mechanism. 

^*67*^ *Ga-Citrate*. ^67^Ga-citrate almost exclusively binds to transferrin in the circulation, a key protein that is responsible for transporting iron between cells and into cells. Transferrin is a *β*-globulin with a molecular weight of 77000, capable of binding two gallium ions simultaneously. Of its two forms, the level of iron-free structure apotransferrin usually exceeds the iron-bound-transferrin in a ratio of ~2 : 1. The normal concentration of apotransferrin in blood is in the order of 40 *μ*M, and this level easily accounts for the number of ^67^Ga-citrate molecules to achieve complete binding. Blood clearance occurs slowly, with 20% of the injected dose remaining in the blood at 24 hours, 10% at 48 hours, and 5% at 72 hours. The abdominal organs of liver, spleen, and kidneys retain ~10% of the initial injected radioactivity, plus ~3% in bone, and over the next few hours there is significant uptake in the bowel. After seven days ~26% of the injected dose has been excreted in urine and ~9% in stools. When there is high iron saturation, the lower level of apotransferrin results in less transchelation from ^67^Ga-citrate, and hence more gallate ions. Under this condition, the biodistribution alters from soft tissue toward bone uptake, in conjunction with more rapid blood clearance and increased urine excretion. It has been postulated that the ^67^Ga isotope binds to leukocytes that migrate to the inflammatory site, or to leukocytes already present at the site. The ^67^Ga-transferrin interaction with leukocytes is not clearly understood but it has been suggested that the internalized ^67^Ga(III) binds to cytoplasmic lactoferrin [5]. Neutrophils contain ample lactoferrin, and this is a common protein found in abscess fluid. When bacteria invade the inflammation site, other retention mechanisms may ensue where ^67^Ga-transferrin provides isotope to more avid ligands such as ferritin or siderophores expressed by microbes.

^*18*^ *FDG.* ^18^FDG is normally distributed to metabolically active organs such as the brain or heart, and also to smaller focal areas of soft tissue activated by an inflammation, pathogenic invasion, or cancer. This radiopharmaceutical was designed as a mimic of glucose, for uptake by active normal cells, inflammatory, or tumour cells with a high demand for sugars, entering into the glycolysis pathway. The structural difference between ^18^FDG and glucose is the presence of a C–F bond in the former molecule, that is not recognized by the hexokinase enzyme in step 1 of the glycolytic reaction sequence. In the first step glucose is phosphorylated at the 6-hydroxy position with hexokinase, then the product is converted in step 2 by ring contraction to fructose 6-phosphate with phosphoglucose isomerase. When ^18^F replaces the hydroxyl group at the 2-position on the glucose ring (i.e., ^18^FDG), step 1 proceeds but step 2 is prevented. The PET isotope subsequently becomes trapped and retained in cells, thereby allowing scintigraphic detection of local metabolism. The reasons for this may be attributed to intracellular enzymes unable to dock with ^18^FDG in the appropriate orientation to initiate step 2, or the C–F bond is not recognized and therefore left intact. It was shown that ^18^FDG is not phosphorylated by hexokinase (i.e., does not enter glycolysis) but is preferentially excreted rapidly from the body, a property resulting in nuclear images with very high target to background ratios [26]. In contrast, glucose participates in resorption from urine to plasma via an active transport process across the renal tubule, rather than its excretion. Resorption succeeds with glucose because it contains the prerequisite 2-hydroxyl group that initiates active transport [27], but fails with ^18^FDG that contains a different functional group at the 2-position.

^99*m*^ *Tc-Tin Fluoride Colloid-Leukocytes*. When ^99m^Tc-tin fluoride colloid-leukocytes in whole blood are injected back into the patient, high liver and spleen uptake ensues, in a whole body distribution that is almost identical to ^99m^Tc-tin fluoride colloid (i.e., typical of reticuloendothelial uptake). All of the radioactivity in the dose is associated with cells, and these ^99m^Tc-cells would be expected to give a different* in vivo* distribution compared to free radiocolloid. One explanation concerns the high percentage (>70%) of ^99m^Tc-tin fluoride colloid-erythrocytes in the* ex vivo* dose, they behave differently* in vivo* such that erythrocytes detach from the weakly-bound radiocolloid, allowing particles to deposit in these organs. Possible mechanisms of detachment include further opsonization from excess opsonins in the circulation, high shear at vessel walls during turbulent blood flow, or red cells distortion to permit transit through finer lung capillaries. There are nevertheless ~11% ^99m^Tc-neutrophils in a patient dose [16], delivered by blood flow near the inflammation/infection, that subsequently migrate to the extravascular site to participate in local activities. 

^99*m*^ *Tc-HMPAO-Leukocytes.* After patient administration of ^99m^Tc-HMPAO-leukocytes, a low level of ^99m^Tc-HMPAO is released* in vivo*, resulting in undesirable accumulation of radioactivity in the gastrointestinal and urinary tracts. In a patient dose there are a higher number of ^99m^Tc-HMPAO-granulocytes than ^99m^Tc-tin-colloid-leukocytes, indicating more ^99m^Tc-HMPAO-cells are available to participate in the immune response. 

^*111*^ *In-Oxine-Leukocytes.* ^111^In-oxine-leukocytes are also prepared* ex vivo* using a leukocyte population isolated from whole blood free of transferrin. Once the radioactive cells are administered to patients, there is an exponential clearance from the blood with a half-life of 5–10 hours, resulting in a final uptake of 20% by the liver, 25% by the spleen, 30% by bone marrow, and 15% in other nontarget organs. There is very low excretion of ^111^In activity in the urine or faeces. ^111^In-leukocytes are viable and they engage in normal cell traffic around the body. As with ^99m^Tc-HMPAO-leukocytes, ^111^In-labeled leukocytes or granulocytes are delivered to the infectious/inflammatory site for local participation. 

^99*m*^ *Tc*-*Besilesomab*. Similar to ^99m^Tc-sulesomab, ^99m^Tc-besilesomab also recognizes the NCA-95 epitope on the granulocyte membrane and tumours expressing CEA. The Product Information Sheet states besilesomab has no effect on activation of complement, granulocyte function, or platelets, and the most commonly reported adverse reaction is developing HAMA in 14% of patients. Blood clearance follows a biphasic kinetic function, an early phase of 0–2 hours and a late phase of 5–24 hours. At 6 hours after injection (pi), 2% of the injected dose is in the liver with 3% in the spleen, the liver uptake is maintained for 24 hours yet spleen uptake decreases to 2%. Fourteen percent of radioactivity is excreted in the urine during 24 hours. ^99m^Tc-sulesomab radiolabels granulocytes and granulocyte precursors* in vivo*, possibly at the local inflammatory site where the immune response is emphasized.

### 2.4. Preclinical Radiotracers-Types, Radiolabeling Methods, and Biosistribution

Preclinical radiotracers are primarily radiochemicals that are evaluated on the bench and in animal models of infection or inflammation, although some clinical investigational agents have been tested in humans (i.e., ^99m^Tc-ciprofloxacin, ^99m^Tc-interleukin-8). These radiotracers are broadly categorized as either small synthetic molecules (<1000 MWT) or macromolecules (>1000 MWT) of biological origin and cells. The design of a radiotracer usually depends on the nonradioactive part of the structure to influence its distribution* in vivo* (Table 4)—the cold part is a known participant in an inflammatory step or it interacts with a pathogen directly. Synthetic radiotracers are small molecules that target upregulated antigens, proinflammatory molecules, or receptors at an inflammation, radioactive-antibiotics target microbes [28], and radiolabeled-biological-molecules target all of these or leukocytes at the lesion site (Table 5). The literature is abundant with reports surrounding their efficacy in small animals, necessary work before clinical application. The highly efficacious preclinical agents are promising because inflammatory reactions occurring* in vivo* can be imaged during the early phase when anatomical changes are not apparent, and this is a major advantage over other radiological imaging modalities. The main criteria for a useful diagnostic agent of infection/inflammation can be summarized as the: (i) radiotracer is prepared efficiently in a simple operation; (ii) focus can be visualized within a few hours after injection with a high target-to-background ratio; and (iii) radiotracer participates in cellular/biochemical pathways of the inflammatory response, without changing the response. Even if a preclinical radiotracer does not achieve clinical investigational status, the knowledge base is continuously growing with information that can potentially translate into new molecules or develop into new methodologies and strategies.

#### 2.4.1. Synthetic Molecules

*N-Formyl-methionyl-leucyl-phenylalanine Chemotactic Peptides.* Chemotactic peptides can bind isotopes such as ^99m^Tc and ^111^In readily via a conjugator group such as DTPA or HYNIC, although isolation of the final product depends on a HPLC purification step that is less convenient than an instant cold kit. N-Formyl-methionyl-leucyl-phenylalanyl-lysine (fMLFK) is a bacterial product known to initiate leukocyte chemotaxis by binding to high affinity receptors on the membrane of polymorphonuclear neutrophils (PMNs) and mononuclear phagocytes, cells that concentrate at an infectious/inflammatory site. The conjugator group is bonded to a derivatized lysine in the molecule, at a location in the structure that is distant from the biologically active moiety. Receptor binding studies with ^111^In-DTPA-fMLFK showed this tracer exhibited high affinity for human PMNs and in high potency: 3 nM concentration produced 50% of a maximal response. In the same study that examined* E. coli *infected muscle in rats [29], the target to background ratio (T : B) of 3 peaked at 60 minutes pi. The HYNIC derivative ^99m^Tc-HYNIC-fMLFK was found to have a high affinity and potency for PMNs* in vitro*, contrary to the analogue HYNIC-fMLF-OMe—a control molecule in which HYNIC is attached to the terminal amino group of methionine [30]. The biodistribution data for ^99m^Tc-HYNIC-fMLFK in rabbits showed this tracer was retained in infected (*S. aureus*,* E. coli*) and inflamed (zymosan) thigh muscles with T : B ratios within a range of 3-4, yet superior to the T : B ratio of ~1 obtained with ^99m^Tc-HYNIC-fMLF-OMe at 4 hours pi. The authors concluded that ^99m^Tc-HYNIC-fMLFK was retained at the infectious or inflammatory site by means of a specific receptor binding mechanism, provided there is sufficient cellular infiltration. These potent radiolabeled-chemotactic peptides can induce transient granulocytopenia, and this undesirable side effect has subsequently impeded their clinical application.

^*68*^ *Ga-Citrate*. ^68^Ga-citrate is easily prepared in >98% yield by reacting ^68^GaCl_3_ with citric acid. The positron-emitting starting material ^68^GaCl_3_ is eluted from a ^68^Ge/^68^Ga-generator, the cation is trapped on an ion exchange column and then washed off in a bolus of citric acid buffer solution [31]. An expected mimic of ^67^Ga-citrate, ^68^Ga-citrate was more recently recruited for clinical investigation in patients with suspected osteomyelitis and discitis. The PET tracer resulted in good diagnostic images, with a sensitivity of 100%, specificity of 76%, and an overall accuracy of 90%; the authors suggested that ^68^Ga-citrate should be compared with ^18^FDG for bone infection [32]. 

^*68*^ *Ga-Siderophores*. Iron is a vital element needed for many cellular processes and by almost all living organisms. Prokaryotes use special mechanisms to acquire ionic iron using their own siderophore molecules or they utilize siderophores released by other microorganisms [33]. Currently known types of siderophores include the catecholates, hydroxamates, and hydroxycarboxylates, which are specific for Fe(III). Ga(III) has similar chemistry to Fe(III), and this has been exploited in radiopharmaceutical chemistry to label molecules such as ferrioxamine (FOXE) and triacetylfusarinine C (TAFC) with the ^68^Ga isotope [34]. TAFC is a common siderophore of many fungal species, and FOXE is mainly produced by actinomycetes and other bacteria. Based on the evidence that such molecules are upregulated at an infectious site, and also scavenged by the microbe from its immediate environment, ^68^Ga-TAFC and ^68^Ga-FOXE were investigated with* A. fumigatus in vitro *[34], as well as* in vivo* using rodent models of lung infection (*A. fumigatus*,* S. aureus*) versus sterile inflammation (turpentine) [35]. In the earlier study [34], ^68^Ga-TAFC and ^68^Ga-FOXE were lowly protein-bound in human serum, both were rapidly taken up by* A. fumigatus* in iron deficient medium, and uptake could be blocked with excess ferri-siderophore* in vitro*. The biodistribution images for ^68^Ga-TAFC and ^68^Ga-FOXE in* A. fumigatus*-infected rat lung showed this tracer was retained in the organ with T : B ratios of 6 and 7, respectively, at ≤30 minutes pi (standard uptake values were ≤1). The authors concluded that further studies were required to distinguish the better of two radiotracers, that nevertheless showed high promise. The following work [35] showed the* in vitro* uptake of ^68^Ga-TAFC and ^68^Ga-FOXE differed between fungi, according to the presence or absence of iron in the medium.* Mycobacterium smegmatis* and three other types of bacteria did not bind either radiotracers ± iron present, except when ^68^Ga-TAFC was incubated with* S. aureus *in an iron deficient medium. Both radiotracers showed rapid focal accumulation in* A. fumigatus* infected rat lungs; there was uptake at the turpentine inflammation site, but none by the* S. aureus* abscess* in vivo*. ^68^Ga-siderophores activity at the sterile inflammation was attributed to nonspecific extravasation. The authors concluded that both radiotracers had potential application for imaging invasive aspergillosis, ^68^Ga-TAFC had higher specificity for* A. fumigatus in vitro* whereas ^68^Ga-FOXE gave slightly superior PET images in the pilot evaluations, in advance of the intended clinical studies.

*Leukotriene Agonists.* Leukotriene (LT) B4 or 5,12-dihydroxyeicosa-6,8,10,14-tetraenoic acid is produced by leukocytes in response to inflammatory mediators, a molecule synthesized from arachidonic acid via the 5-lipoxygenase pathway. LTB4 is a secondary chemoattractant secreted early in the inflammatory process, and a signal-relay molecule that regulates neutrophil chemotaxis to formyl peptides [36] which are released at the core sites of inflammation. It also stimulates leukocyte vascular adhesion, transendothelial migration, and the release of lysosomal enzymes [37]. Recently the radiolabeled LTB4 antagonist ^111^In-DPC11870 was prepared and evaluated for binding to white blood cells* in vitro*, and* in vivo* in rabbits with an* E. coli* thigh muscle infection. Ninety-five percent of the radiotracer was found to be cell associated; there was high uptake in the abscess on the images as well as bone marrow uptake. The results showed ^111^In-DPC11870 accumulated at infectious foci because of LTB4 receptors expressed on activated hematopoietic cells that subsequently migrated from the marrow to the infection site [38]. A popular animal model of experimental colitis uses 2,4,6-trinitrobenzenesulfonic acid (TNBS) in ethanol, a chemical that insults the colonic mucosa after rectal instillation. ^111^In-DPC11870 was used in rabbits with a TNBS-colitis, the inflamed lesion was clearly delineated even 1 hour after injection, a T : B ratio of ~12 was determined at 6 hours pi, and the images were better than those obtained from ^18^FDG and ^99m^Tc-granulocytes [39]. The same researchers also looked at radiolabeling the LT agonist with other isotopes such as ^99m^Tc via the HYNIC conjugator [40], and ^18^F via [^18^F]-*p*-trimethylammoniumbenzaldehyde triflate (FB) [41]. ^99m^Tc-HYNIC-MB88 showed much less bone marrow uptake than ^111^In-DPC11870, attributed to the HYNIC moiety in the structure, and it gave a T : B ratio of 15 at 8 hours pi. The product ^18^F-FB-HYNIC-MB67 was synthesized in moderate radiochemical yield, by formation of a hydrazone bond between the aldehyde group in ^18^F-FB and an amino group in HYNIC (-MB67). Even though this PET radiotracer was prepared with low specific activity, a T : B ratio of ~22 was achieved at 4 hours pi in rabbits with an* E. coli* muscle infection; good images were acquired early at 2 hours pi and these only improved with time.

#### 2.4.2. Antibiotics

An emerging area of research involves radiolabeled antibiotics, mainly because the cold antibiotic has a known microbial action, and some are commercially available as prescription medicines. ^99m^Tc has been the primary isotope so far for radiolabeling quinolones, cephalosporins, phosphopeptides, glycopeptide, aminoglycoside, aminocoumarin, and an antifungal triazole. The ^99m^Tc-antibiotics in Table 4 are prepared easily in a cold kit format and predominantly within 30 minutes. The more versatile stannous salts are commonly used as reductants, although potassium borohydride and sodium dithionite are also important. An interesting reagent tricarbonyl-^99m^Tc was used to efficiently label novobiocin, conveniently prepared by heating ^99m^Tc-pertechnetate in an IsoLink vial containing potassium boranocarbonate (Mallinckrodt Medical) at 100°C for 20 minutes. For any antibiotic molecule, the absence of a conjugating group in the structure means the isotope coordinates directly to available donor atoms, and the radiometal can interfere with the expected pharmacological action. An extra purification step is a practical disadvantage but necessary to remove unwanted impurities when the radiolabeling efficiency is low, as per example of enrofloxacin. Once the ^99m^Tc-antibiotic is* in vivo*, the magnitude of a target to background ratio is influenced by the binding affinity of the radiotracer to the bacterial surface or intracellular components, as well as the level of cold antibiotic in the dose. An excess of cold antibiotic makes the radiotracer less efficacious because there is competitive binding, or the pathogen is killed at the infectious site. For example, ^99m^Tc-kanamycin was highly bound to bacteria* in vitro*, particularly when very low kanamycin was present in the dose. Furthermore, it is important in any study of a new radiotracer to compare it with a permeation capillary marker or other marker in the same animal model, to confirm whether a retention mechanism rather than just blood flow occurs at the infectious site.

*Quinolones.* Antimicrobial quinolones were introduced into clinical practice in the early 1960s, commencing with naladixic acid. This new class of antibiotics were chemically synthesized for the first time in a laboratory procedure that superseded the regular laborious method of isolating molecules from mould and fungi. Ciprofloxacin was the first antibiotic to be crudely radiolabeled with ^99m^Tc in the presence of formamidinesulfinic acid reductant at 100°C for 10 minutes, to give >40% product [42]. This radiolabeling procedure was later improved.* In vitro* binding studies of ^99m^Tc-ciprofloxacin (Infecton) with* S. aureus*,* P. aeruginosa, *and* E. coli* found 58%, 50%, and 44% binding, respectively. The mechanism of action of this molecule and other antibiotic quinolones involves their interaction with bacterial DNA gyrase (a type II topoisomerase) that prevents DNA uncoiling and subsequent DNA synthesis. In a preclinical study involving rats with sterile and infectious intramuscular lesions, ^99m^Tc-ciprofloxacin and ^99m^Tc-enrofloxacin showed peak uptake after 1 hour pi in* S. aureus *and* C. albicans* focal sites, followed by a decrease over the next 4 hours [43]. Both radiotracers showed low binding (~5%) to bacteria, irrespective if the bacteria were dead or alive, and there was no influence of excess cold antibiotic in the dose. Neither tracer could discriminate between infection and sterile inflammation. A clinical study of 51 patients with chronic orthopaedic infections found Infecton to be useful for detecting infectious foci in bones and joints, in which the authors proposed that this radiotracer had advantages over ^99m^Tc-HMPAO-leukocyte scintigraphy [44]. The biodistribution data for ^99m^Tc-pefloxacin showed that this tracer was retained in* S. aureus* infected thigh muscle of rats, with a peak T : B ratio of 5 and it was superior to that obtained with Infecton [45]. Other noted quinolones recently investigated as antibiotic radiotracers are ^99m^Tc-difloxacin in* S. aureus* infected rats [45], ^99m^Tc-sitafloxacin in* S. aureus* infected rats [46], ^99m^Tc-sparfloxacin in* S. aureus* infected rats [47], ^99m^Tc-lomefloxacin and ^99m^Tc-ofloxacin in* S. aureus* infected rats [48], and ^99m^Tc-moxifloxacin in* E. coli* infected rabbits [49].

*Cephalosporins.* Cephalosporin compounds were first isolated in cultures of* Cephalosporium acremonium* obtained from a Sardinian sewer in 1948. Since then, there have been four generations of these pharmaceuticals employed in the clinic. The cephalosporins are *β*-lactam antibiotics that function against bacteria by disrupting the synthesis of the peptidoglycan layer and hence the structural integrity of a cell wall. Penicillin-binding proteins bind to a D-Ala-D-Ala sequence at the end of muropeptides (peptidoglycan precursors), to crosslink the peptidoglycan during a final transpeptidation step of its synthesis. Beta-lactam antibiotics competitively bind to the D-Ala-D-Ala site, thereby interfering with the crosslinking step. Unlike ceftizoxime and cefoperazone, cefuroxime axetil is a semisynthetic cephalosporin, a prodrug which owes its* in vivo *bactericidal activity to the release of the active compound cefuroxime (Table 4). The ^99m^Tc-labeling procedure for all three antibiotics was easily achieved with efficient conversion. In separate antimicrobial activity experiments, ^99m^Tc-ceftizoxime was compared to ceftizoxime by measuring the inhibition halo on cultured plates. The authors found antibiotic activity was preserved; they also noted a difference of 10% less activity when the radiometal was bound [50], despite the ^99m^Tc-ceftizoxime dose containing ceftizoxime. A low T : B ratio of 2 was maintained over 1–6 hours in* E. coli* infected rat muscle in the same study. ^99m^Tc-cefoperazone was examined in* S. aureus* infected thigh muscles of rats; it yielded a peak T : B ratio of ~5 at 45 minutes and a gradual decline thereafter [51], versus a ratio of 2.5 at 4 hours pi by ^99m^Tc-cefuroxime axetil in the same model [52]. Unfortunately none of these studies included a comparison of their radiotracer to a blood flow marker, bacterial-binding assays were not always conducted, and therefore an* in vivo* retention mechanism remains unproven. More recently the radiolabeled cephalosporin ^99m^Tc-ceftriaxone has also been investigated in* S. aureus* infected mice with mixed success [53].

**Figure 2:**
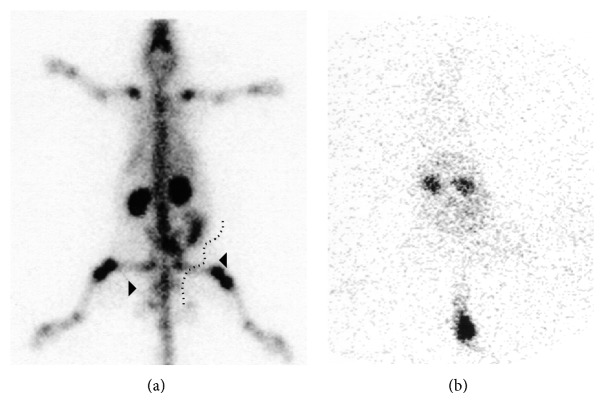
Four-hour whole body images of rats with a TNBS-colitis, injected with (a) ^99m^Tc-alafosfalin (annotated line represents the descending colon plus increased uptake ◂ in distal colon) and (b) ^99m^Tc-DTPA.

*Phosphopeptides.* Phosphomycin was discovered in 1969 in soil bacteria cultures of* Streptomyces fradiae,* and then two years later it was manufactured on a large scale in Spain. It is now produced by chemical synthetic methods. Phosphomycin enters the bacterial cell through the glycerophosphate transporter and then inhibits bacterial cell wall synthesis. It directly inhibits UDP-*N*-acetylglucosamine-3-enolpyruvyl-transferase by alkylating an active cysteine residue that would otherwise catalyze phosphoenol-pyruvate binding to UDP-*N*-acetylglucosamine, in a key step of peptidoglycan biosynthesis. This pyruvate moiety provides a linker that bridges the glycan and peptide portion of peptidoglycan comprising the cell wall. Alafosfalin or (S)-alanyl-1-aminoethylphosphonic acid is broad spectrum antibiotic that selectively inhibits cell wall synthesis in both gram positive and gram negative bacteria. This dipeptide antibiotic is actively transported intracellularly by stereospecific permeases, an amide hydrolysis reaction by aminopeptidase then affords L-1-aminoethylphosphonic acid which in turn inhibits alanine racemase. Alanine racemase is a key enzyme required to synthesize peptidoglycans that comprise prokaryote cell walls. The ^99m^Tc-labeling procedure for both antibiotics was easily and efficiently achieved using stannous ions. In* S. aureus* infected mouse muscle, ^99m^Tc-phosphomycin gave T : B ratios of 3-4 during 1–24 hours, compared to the blood flow marker ^99m^Tc-DTPA that gave T : B ratios of 2–5 during the same time period [54]. The same study of* E. coli* in mouse model gave ^99m^Tc-phosphomycin T : B ratios of 3-4 during 1–24 hours, but no bacterial binding assay was performed to prove the retention mechanism. This radiotracer was limited by lower T : B ratios than ^67^Ga-citrate and resulted in diffuse rather than focal uptake at the infectious lesion site. For ^99m^Tc-alafosfalin, ≤10% was bound to* S. aureus in vitro* and higher binding was achieved when the amount of alafosfalin in the dose was lower. This relationship did not apply to ^99m^Tc-DTPA that uniformly resulted in 1% bacterial binding [55]. In* S. aureus* infected rat muscle, ^99m^Tc-alafosfalin gave a T : B ratio of 3 at 1 hour that increased to 4 at 4 hours, compared to ^99m^Tc-DTPA with T : B ratios of 3 at 1 hour and then 2 at 4 hours, or ^99m^Tc-stannous colloid-rat leukocytes with T : B ratios of 12 at 1 hour and then 20 at 4 hours. Similar results were observed when ^99m^Tc-alafosfalin was examined in* S. aureus* infected mouse muscle, yielding T : B ratios of 3 at 1 hour and then 4 at 4 hours versus ^67^Ga-citrate with T : B ratios of 2 and then 4 at the same time points, respectively [56]. ^99m^Tc-alafosfalin was comparable to ^67^Ga-citrate but inferior to ^99m^Tc-stannous colloid-leukocytes for imaging a focal infection in rodents. The authors suggested that this radiotracer may be useful for imaging abdominal and soft tissue infection because of its low uptake after 1 hour pi. ^99m^Tc-alafosfalin was also investigated in rats with TNBS-colitis, it yielded T : B ratios of 3 at 1 hour then 4 at 4 hours, versus ^99m^Tc-DTPA with T : B ratios of 2 then 1 at the same time points respectively [57] (Figure 2). The quick cold kit preparation had practical advantages over conventional cell labeling methods.

*A Glycopeptide, Aminoglycoside, Aminocoumarin, and an Antifungal Triazole.* Vancomycin is used as a prophylactic antibiotic to treat infections caused by gram positive bacteria. The diagnostic value of ^99m^Tc-vancomycin was investigated in* S. aureus* infected rat muscle, giving a T : B ratio of 5 at 1 hour pi, and a T : B ratio of 1 in turpentine inflamed rat muscle at 1 hour [58]. When the results were taken together, the ratio of 1 from inflamed muscle indicated that bacteria must be present at the site for ^99m^Tc-vancomycin uptake. Kanamycin is an antibiotic molecule that is taken up intracellularly and then interacts with the 30 S subunit of prokaryotic ribosomes. It induces substantial amounts of mistranslation and indirectly inhibits translocation during protein synthesis. ^99m^Tc-kanamycin was found to have 40–50% binding to* S. aureus in vitro* versus 10% binding of ^99m^Tc-DTPA to the same microbe. The level of kanamycin in the radioactive dose was 2–50 *μ*g [59]. Biodistribution in rats and rabbits achieved maximal T : B ratios ranging over 2-3, that were maintained over 4–24 hours. Novobiocin and other aminocoumarins are inhibitors of bacterial DNA gyrase by binding to the enzyme GyrB subunit. More potent than fluoroquinolones, aminocoumarins competitively inhibit the ATPase reaction catalysed by GyrB for energy transduction. The dithiocarbamate derivative of novobiocin was radiolabeled with [^99m^Tc(CO_3_)(H_2_O)_3_]^+^ in high yield without the need to purify the final product. ^99m^Tc-novobiocin distribution in* S. aureus* infected rat muscle achieved a maximal T : B ratio of 8 at 90 minutes, it subsequently decreased, yet in a sterile inflammation the T : B ratio was ≤1 for up to 120 minutes, indicating radiotracer specificity for bacteria [60]. These results concurred with observations made by the authors in the same study that employed rabbits with intramuscular* S. aureus *infections.

Fluconazole is a pharmaceutical used to treat fungal infections of* C. Albicans* or* A. Fumigatus*, that can result in patients following anticancer therapy, organ transplantation or AIDS. The ^99m^Tc-analogue was investigated in a rodent model to distinguish fungal from bacterial infection, and infection from sterile inflammation [61].* In vitro* binding studies found that ^99m^Tc-fluconazole preferentially bound to* C. Albicans* (38%) over* A. fumigatus* (18%), or human leukocytes (12%),* K. pneumoniae* (6%), and* S. aureus* (3%). Further evaluation in infected mouse muscle gave T : B ratios over 1–4 hours, as 3-4 for active* C. albicans* and <1 for dead* C. albicans*. In neutropenic mice, the T : B ratios in thigh muscle over 1–4 hours were 1-2, for* A. fumigatus* and sterile inflammation. The authors also reported this radiotracer was capable of distinguishing a fungal infection from a bacterial infection or inflammation, and it was particularly suited for the* C. albicans *fungus.

#### 2.4.3. Molecules of Biological Origin

*White Blood Cells*. There is an incentive to replace ^111^In or ^99m^Tc in the radiotracer structure with a *β* ^+^-emitting isotope because of potentially higher image resolution and faster diagnoses, and this idea has led to the preparation of ^18^FDG-leukocytes. The ^18^FDG-labeling efficiency of autologous leukocytes is dependent upon the concentration of glucose in the reaction medium; higher yields are obtained when extracellular glucose is lower, and 80% of radiogranulocytes are viable for up to 4 hours in platelet poor plasma [62]. ^18^FDG-leukocytes were examined in rat muscle that was infected (*P. aeruginosa, E. coli*) or inflamed (turpentine) [63]. The more important finding was the higher uptake by thigh muscle infections over normal muscle, clearly visible on the images. The T : B ratios were twice higher than ^18^FDG, and these findings allowed the authors to conclude that the release of ^18^FDG from ^18^FDG-leukocytes at the lesion focus did not fully explain the observed uptake. Clinical investigations with ^18^FDG-leukocytes have suggested that this radiotracer is comparable to ^111^In-leukocytes for patients with suspected infection in terms of diagnostic accuracy, but possibly not for patients with suspected osteomyelitis [64]. Two drawbacks with ^18^FDG-leukocytes are the rapid loss of ^18^FDG label from white cells* in vivo*, and the short half-life of ^18^F-isotope prevents delayed imaging that may consequently also reduce its specificity [65].

**Figure 3:**
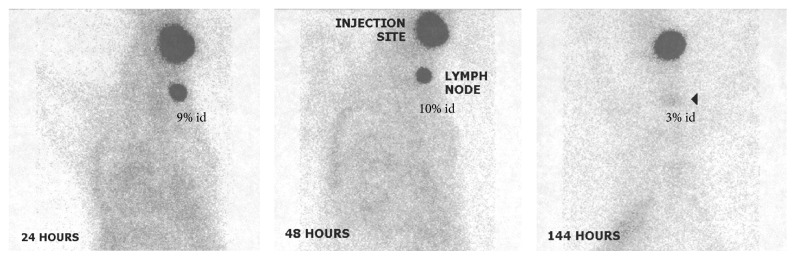
Anterior static images of a sheep taken at 24, 48, and 144 hours after a subcutaneously injected dose (id) of ^111^In-dendritic cells in the neck and migration to the nearest lymph node (◂).

Other white cells of the immune system such as dendritic cells and lymphocytes have also been radiolabeled, with the aim to further understand cellular and molecular mechanisms underlying infectious diseases, or inflammation such as organ allograft rejection. Radiolabeling of dendritic cells was more successful with ^111^In-oxine (36%) than ^99m^Tc-HMPAO (17%), and even after their purification the ^111^In-dendritic cells were viable and stable in contrast to ^99m^Tc-HMPAO-dendritic cells [66]. This sheep study showed that the subcutaneous injection of naïve ^111^In-dendritic cells resulted in 10% uptake in the supraclavicular lymph nodes after 48 hours (Figure 3). These results support the hypothesis that dendritic cells do traffic to local lymph nodes for the purpose of encountering resident T lymphocytes there, before chemotactic recruitment of circulating leukocytes to the antigen source. Autologous lymphocytes were also radiolabeled with ^111^In-oxine; the ^111^In-lymphocytes were administered to kidney-graft patients to diagnose acute organ graft rejection [67]. When acute graft rejection was present, it was visible on the images that the graft : contralateral iliac fossa ratio increased; whereas the spleen : graft ratio decreased. An improvement after the rejection crisis occurred, parallel to the graft : contralateral iliac fossa ratio. The authors concluded that ^111^In-lymphocytes may be useful in the diagnosis of acute kidney graft rejection.

*Macromolecules*

**Figure 4:**
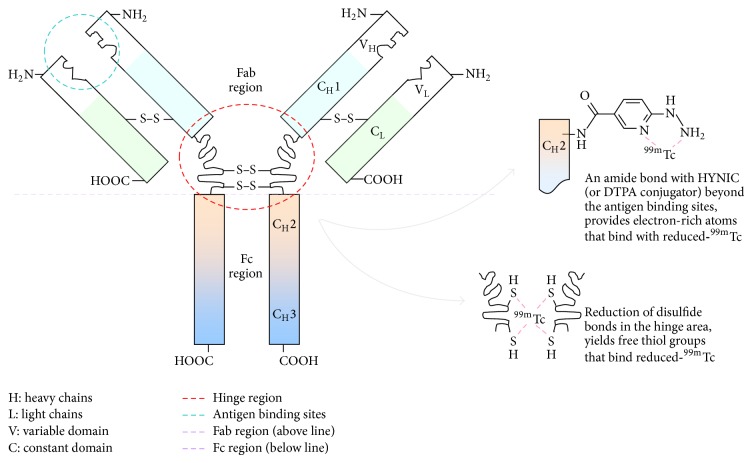
An annotated diagram of IgG depicting the antigen binding sites and isotope binding sites.

*Immunoglobulins*. Immunoglobulins (Ig) or antibodies (Figure 4) are large Y-shaped proteins produced by B-cells for the purpose of identifying and neutralizing antigens as part of host immunity. In placental mammals, the five known antibody isotypes are IgA, IgD, IgE, IgG, and IgM. IgA has a dimeric structure, and of the two subtypes IgA1 is found in serum, whereas IgA2 is predominantly found in secretions and secretory lymphoid tissues. IgD and IgE are both monomers, each with one subtype. IgG is an abundant immunoglobulin found in blood and extracellular fluid; it has a monomer structure, and there are four subtypes IgG1, IgG2, IgG3, and IgG4. These subtypes differ by their binding affinities to the Fc receptor on phagocytic cells and the extent of activating the complement system. Other IgG functions include mediated binding of pathogens to achieve immobilization and agglutination; coating of pathogen surfaces or opsonization, for recognition and ingestion by phagocytic cells; binding and neutralizing toxins; and mediated proteolysis by binding intracellular TRIM21 receptor and directing marked virions to the proteasome in the cytosol. The largest antibody in the circulation is the pentameric IgM, predominantly produced in the spleen as one subtype, and it is the first antibody to appear after sudden exposure to an antigen. Almost all radiolabeled-antibody laboratory experiments have utilized cloned IgGs, and when the four subtypes comprise the raw material it is described as polyclonal (pc) IgG. A monoclonal antibody (mAb) is derived from one cell line. Usually a new radiolabeled-antibody is examined in rodent model of infection/inflammation, with comparison to either a “gold standard” agent (i.e., ^67^Ga-citrate or ^111^In-leukocytes), control molecules such as a nonspecific similar-sized antibody, or a nonspecific protein that is solely a blood flow marker (i.e., ^99m^Tc-HSA).

**Figure 5:**
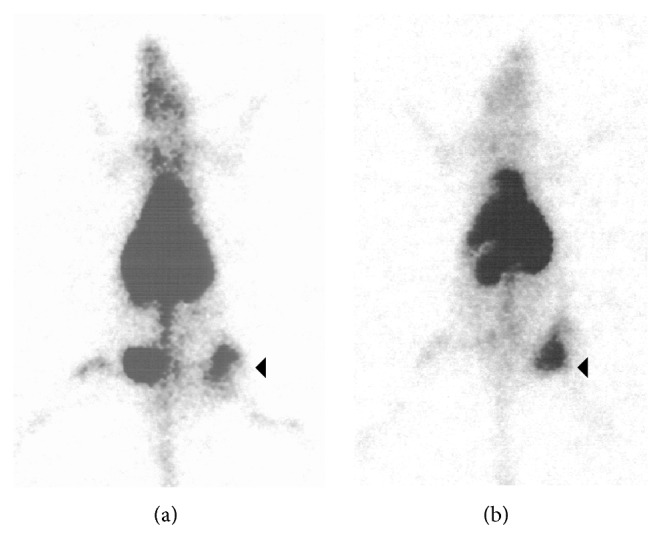
Whole body images of rats with* S. aureus *infections (◂) in left thigh at 4 hours pi with (a) ^99m^Tc-hIgG and (b) ^99m^Tc-rIgG.

When pcIgG is reacted with DTPA-anhydride, one amino group on the immunoglobulin forms a covalent bond per DTPA molecule beyond the antigen binding sites, making it available to coordinate with a radiometal such as ^111^In(III). No reductant is required to achieve a radiolabeling efficiency of >95% ^111^In-DTPA-IgG when 2-3 DTPA conjugating groups are attached per IgG molecule [68]. In a study of rats with* S. aureus* muscle infections, ^11^In-DTPA-IgG(^14^C) showed peak uptake in the abscess with a T : B ratio of 10 after 2 hours pi, followed by a gradual decrease to 9 at 6 hours pi and then 6 at 24 hours pi, respectively [43]. This dual isotope tracer was used to demonstrate a partial dissociation of ^111^In (but not ^14^C) from the molecule at the abscess as well as in other tissues. Possible explanations offered by the authors included proteolysis in the interstitial fluid space at the inflammation site, and transchelation to local transferrin or other endogenous molecules. ^99m^Tc-(human)IgG was examined against a control antibody ^125^I-TNT-1, ^99m^Tc-HSA, and ^67^Ga-citrate in mice with an infection (*S. aureus*) or an inflammation (turpentine) [69]. Results for the infection model showed T : B ratios at 4 hours pi of 4 (^99m^Tc-hIgG), 4 (^125^I-TNT-1), 4 (^99m^Tc-HSA), and 5 (^67^Ga-citrate), and for the inflammation model T : B ratios at the same time point were 3-4 for all radiotracers. The authors used autoradiographic data to support a conclusion that the radioactive proteins enter into the interstitial space due to increased capillary permeability, however there was little elaboration about any retention mechanism. Increased blood flow to the inflammatory site explained some of the early radioactive uptake, and only if the radiotracer was bound to resident immune elements could it be visible as accumulated uptake in the delayed scans. A hIgG cold kit formulation was commercially available for many years, for direct labeling to the immunoglobulin with ^99m^Tc. Direct labeling of the antibody can be achieved after modifying the hinge area of the structure, by reducing disulfide bonds into free thiol groups to coordinate radiometal (Figure 4). Usually a stannous salt of MDP, tartrate, and so forth facilitates ^99m^Tc-pertechnetate reduction. The efficacy of ^99m^Tc-hIgG as the control molecule versus ^99m^Tc-rat(r)-IgG in a rat model of* S. aureus* muscle infection was previously examined [70]. Even though ^99m^Tc-rIgG was prepared from allogeneic immunoglobulin, the results for this radiotracer showed a T : B ratio of 13 at 4 hours pi, superior to ^99m^Tc-hIgG ratio of 8 at the same time point, and uptake was clearly visible on the whole body images (Figure 5). The authors suggested the Fc portion of hIgG contained a different glycosylation pattern (oligosaccharides with N-acetyl neuraminic acid) than rIgG (oligosaccharides mixed with N-acetyl neuraminic acid and N-glycolylneuraminic acid), and this difference affected the binding specificity to Fc*γ* receptors on monocytes, macrophages and neutrophils residing at the infectious site, favouring “endogenous” rIgG. ^99m^Tc-hIgG was examined in a small clinical investigation against ^111^In-granulocytes for the localization of inflammatory bowel disease [71], but it was found to be unsuitable for identifying diseased intestinal segments because of low target to background activity, in contrast to ^111^In-granulocytes that were deemed more suitable.

When 3-succinimidyl-6-hydrazinonicotinate (succ-HYNIC) is reacted with one amino group on the immunoglobulin a covalent amide bond is formed on the 3-nicotinyl position (Figure 4) at the expense of a succinyl group. The HYNIC conjugating group is located beyond the antigen binding site areas, and conditions can be varied to increase the number of HYNIC groups. HYNIC-modified-IgG is purified by size exclusion chromatography before the next step of ^99m^Tc-chelation to hydrazine groups. ^99m^Tc-HYNIC-hIgG is prepared in high yield using either stannous chloride, Mg^2+^, and EDTA [72], or stannous sulphate and tricine [73]. ^99m^Tc-HYNIC-hIgG examined in mice with turpentine-inflamed muscle resulted in a T : B ratio of 6 after 6 hours pi, and the ratio in granulocytopenic rats with* S. aureus* muscle infections was 18 after 24 hours pi. When the rats were subjected to an* A. fumigatus* lung infection, there was >7x greater uptake of ^99m^Tc-HYNIC-hIgG than ^67^Ga-citrate at the lesion site. ^99m^Tc-HYNIC-hIgG was compared to ^111^In-DTPA-IgG in a clinical study of 39 patients with suspected infectious or inflammatory disease [74]. Both radiotracers were equally sensitive (100%) and specific (85%) for detecting of joint prosthesis infection, bone infection, soft tissue/hip infection, spondylodiscitis, fever of unknown origin, or pulmonary sarcoidosis. 

**Figure 6:**
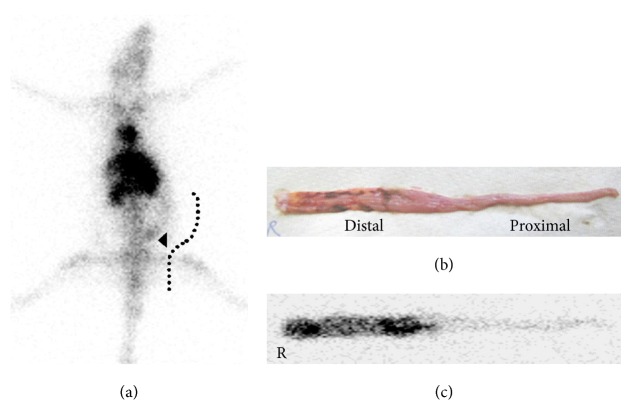
Four-hour ^99m^Tc-infliximab distribution in a rat with TNBS-colitis: (a) whole body image (annotated line represents the descending colon, with increased uptake ◂ in distal colon); (b) resected intestine after TNBS exposure (distal inflammation); and (c) radiotracer distribution in the same resected specimen (R = rectum).

*Monoclonal Antibodies*. The methodology to directly label monoclonal antibodies is the same with any immunoglobulin structure, in which the radiometal is exclusively coordinated to the hinge area. The modified hinge area is sufficiently distant from the V_H_/V_L_ sites in the immunoglobulin, allowing free interaction with the antigen it was designed for. This was shown for ^99m^Tc-infliximab, a chimeric mAb [75 : 25, human : mouse] specific for TNF*α* [70]. In this study reduced-infliximab retained its specificity for the human antigen, and after its reconstitution as a cold kit formulation, ^99m^Tc-infliximab was also specific. Neither of these immunoglobulin molecules could bind rat TNF*α*. Nevertheless, in a rat model of* S. aureus *muscle infection ^99m^Tc-infliximab gave a T : B ratio of 7 at 4 hours pi versus a ratio of 8 with ^99m^Tc-(human)pc-IgG, indicating an immunological binding mechanism at the lesion site that excludes TNF*α* binding. This radiotracer was inferior to ^99m^Tc-leukocytes when examined in a rat model of experimental TNBS-colitis, yielding a T : B ratio of 3 at 4 hours pi (Figure 6) versus the ^99m^Tc-cells ratio of 41 at the same time point [75]. At the clinical level, ^99m^Tc-infliximab was investigated against ^99m^Tc-HMPAO-leukocytes in 10 patients with active Crohn's disease [76]. The authors found that there was comparatively lower bowel uptake and concluded the low TNF*α* concentration at the active site suggested the mechanism of action of a nonradioactive therapeutic dose required more investigation. More recently this diagnostic agent was examined in patients with refractory monoarthritis for therapy decision-making [77]. There was significantly higher uptake seen on the images of joints at 6 hours pi before therapy, compared to posttherapy. The authors concluded this radiotracer could be a useful tool to predict clinical response to intra-articular infliximab treatments.

NeutroSpec or kit for the preparation of ^99m^Tc-fanolesomab, was an FDA-approved product for clinical use in 2004 but it was later withdrawn from the market in 2005 because of a fatal event and a life-threatening cardiopulmonary event [78]. Fanolesomab is a murine IgM monoclonal antibody directed against 3-fucosyl-N-acetyllactosamine, a carbohydrate moiety comprising the CD15 antigen that is expressed by PMNs, eosinophils, and monocytes. ^99m^Tc-fanolesomab was indicated for scintigraphic imaging of patients with equivocal signs and symptoms of appendicitis, by targeting upregulated CD15 antigen at the inflammatory site. The kit formulation that achieved successful labeling contained fanolesomab, maltose, stannous tartrate, succinic acid, glycine, and EDTA. After ^99m^Tc-pertechnetate addition, the reaction vial required heating at 37°C for 30 minutes, and then ascorbic acid solution was finally added to yield ≥90% product. At 35–65 minutes pi of ^99m^Tc-fanolesomab, radioactivity was associated with neutrophils (14–50%) and lymphocytes (~10%) in circulating blood [79]. Neutrophils bound with fanolesomab still retained their adherence and phagocytic functions to* S. aureus* or* E. coli*, as well as their chemotactic ability [79]. In a trial of 49 patients with equivocal appendicitis, the images revealed the inflammation site in all cases with 100% sensitivity, ~80% specificity and a T : B ratio of 2 at 36–40 minutes pi [80]. Since its availability to nuclear imaging clinics, ^99m^Tc-fanolesomab was used in 11000 patients, two of those patients with underlying cardiopulmonary conditions died, although the relationship with NeutroSpec was not resolved at that time [79].

A review of the mAbs literature has revealed there is a more recent emphasis towards commercializing humanized and human mAbs over chimeras or those of murine origin, possibly due to the history of product withdrawals from the market (Table 6). Unfortunately a high proportion of radiolabeled-mAbs were also withdrawn from the market, the major reason was compromised patient safety [81, 82], and to a lesser extent product quality such as problems with raw materials in the product formulation. In the 1980s mAbs were described as “magic bullets” or highly specific vectors for targeting antigens, with promised widespread medical applications. Particularly for diagnostic nuclear medicine, the “magic bullets” have not yet been fully realized. In terms of advancing radiopharmaceuticals however, researchers are directed toward designing molecules that are smaller than immunoglobulins, by incorporating a risk management approach to avoid potential safety and quality problems during preclinical/clinical investigations [83]. The literature is abundant with examples of such radiotracers; some are described below and include proteins, signalling molecules, or peptide fragments that directly participate in immunological pathways.

*E-Selectin, V-CAM, and Other Proteins*. E-selectin (or CD62E) is a 95 kDa glycoprotein and an adhesion molecule expressed on activated endothelium; its upregulation near the inflammation site occurs with cytokines such as interleukin-1*β* and TNF*α*. This molecule binds to receptors on neutrophils, monocytes, T lymphocytes, eosinophils, and basophils. E-selectin mediates the rolling action of these cells along the endothelial surface and also activates other steps in the adhesion process, in a strategy for their migration toward the inflammation site. Peptides with a high affinity for E-selectin have been characterized, including E-selectin-binding peptide (ESbp) that was labeled with ^99m^Tc [84]. HYNIC was used as the conjugate group to prepare (HYNIC)_2_-ESbp, by its reaction with lysine amine groups within the 16 amino acid backbone structure. When the reaction conditions employed a 10 : 1 excess of HYNIC over a long incubation period, the resulting molecule became biologically inactive (control-ESbp). ELISA assays were used to confirm the high, specific binding of ^99m^Tc-(HYNIC)_2_-ESbp (but not control-ESbp) to E-selectin. The authors used an adjuvant-induced rat model of arthritis, by administering a subcutaneous tail injection of* Mycobacterium butyricum*, and assessing the condition by measuring the diameters of ankles some weeks later. At 11, 14, and 24 days, ^99m^Tc-(HYNIC)_2_-ESbp images at 8 hours pi showed a focus of counts in the right ankle, and ROI analyses gave 38%, 65%, and 34% uptake, respectively. The control-ESbp in the arthritis model gave no ankle uptake, and ^99m^Tc-(HYNIC)_2_-ESbp in a normal rat also gave no uptake. These results demonstrated that activated endothelium could be targeted with ^99m^Tc-(HYNIC)_2_-ESbp. In another study, the analogue ^99m^Tc-IMP-178 was investigated for its potential to image pyogenic osteomyelitis in the rat [85]. The highest uptake of the radiotracer in pyogenic bone occurred at 60 minutes pi. The organ assay results yielded a T : B ratio of 2 versus the nonspecific control molecule ^99m^Tc-IMP-100 that also gave a ratio of 2 at the same time. The authors attributed the ^99m^Tc-IMP-178 uptake in acute osteomyelitic lesions to the interaction with activated vascular endothelium.

Vascular cell adhesion molecule 1 (VCAM-1 or CD106) is a protein encoded by the VCAM1 gene in humans. The VCAM-1 gene contains six or seven immunoglobulin domains, and is expressed on both large and small blood vessels only after the endothelial cells are stimulated by cytokines. Upregulation of VCAM-1 in endothelial cells results from increased gene transcription in response to TNF*α*/interleukin-1 activity, and through stabilization of mRNA via interleukin-4 activity. VCAM-1 expression can be sustained for 24 hours. The VCAM-1 protein is primarily an endothelial ligand for VLA-4 of the *β*1 subfamily of integrins and integrin *α*4*β*7, although it has also been found in other cell types such as smooth muscle cells. The VCAM-1 protein mediates the adhesion of lymphocytes, monocytes, eosinophils, and basophils to vascular endothelium. It also functions in leukocyte-endothelial cell signal transduction, suggesting that it may play a role in the development of atherosclerosis and rheumatoid arthritis. Human leukocyte antigen- (HLA-) derived peptides B2702-p and B2702-rp were found to have a high binding affinity for VCAM-1 and they induced immunomodulatory effects* in vivo* [86]. B2702-p and B2702-rp are fragments of the MHC-I molecule B2702, comprised of residues 75–84 and the repeated sequence 84–75/75–84, respectively. Both fragments were ^125^I-labeled in >95% efficiency using chloramine-T, and then examined in normal rabbits versus hyperlipidemic rabbits as a model of artherosclerotic plaques [87]. The authors found mean aortic uptake of ^125^I-B2702-p and ^125^I-B2702-rp was >2x-fold higher in hyperlipidemic rabbits than control rabbits at 30 minutes pi; however the high lung uptake and hepatobiliary excretion of ^125^I-B2702-rp gave way to the preferred ^125^I-B2702-p that was excreted via the kidneys. In the same study other isotopes were used to label B2702-p for animal imaging: ^123^I-B2702-p was prepared using the chloramine-T iodination method with 80–90% efficiency, and ^99m^Tc-B2702-p was prepared using the ^99m^Tc (OH_2_)_3_(CO)_3_ ISOLINK method with >90% efficiency in which the conjugate group linked onto a histidine in the structure. Hyperlipidemic rabbit images showed 2-3x-fold increased aortic uptake of both radiotracers at 3 hours pi versus the control rabbits. The presence of atherosclerotic lesions was confirmed with Sudan IV and autoradiography; the authors concluded that radiolabeled-B2702-p has potential for imaging VCAM-1 in atherosclerosis. More recently the tetrameric-peptide ^18^F-4V was prepared using the fluorobenzaldehyde method [88], it was shown to have high specificity for soluble VCAM-1* in vitro*; in mouse models of myocardial infarction and heart transplantation it gave T : B ratios of 3 and 2, respectively, at 60–120 minutes pi. The authors also used a mouse model of atherosclerosis in the same study, the highly resolved PET images for each model allowed normal tissue to be clearly distinguished from atherosclerotic lesions, the infarct and the heterotopic allograft, in which VCAM-1 expression was increased.

Translocator protein (TSPO) molecule is an 18 kDa protein receptor mainly found on the outer mitochondrial membrane of hemopoietic and lymphatic cells and in other tissues. TSPO binds with high affinity to cholesterol and transports it across the mitochondrial membrane where it is used in steroid synthesis, and has other functions in the iron-transporter system, apoptosis, or immunomodulation after injury. TSPOs have broad actions on immune cells including modulation of oxidative bursts by neutrophils and macrophages, inhibiting macrophage secretion of cytokines, and inhibiting lymphoid cells proliferation. The TSPO binding ligand ^18^F-FEDAC was investigated in rat model of lung injury (a tracheal insult with LPS) [89]. The authors found TSPO expression was elevated in the inflamed lung section and this correlated to the radiotracer uptake as well as the severity of inflammation. Another PET analogue ^11^C-PK11195 was examined in a clinical trial of 32 patients with symptomatic or asymptomatic carotid stenoses, for imaging intraplaque inflammation [90]. From the images T : B ratios of 0.9 for asymptomatic plaques and 1.1 for symptomatic plaques were determined, and there was significant correlation between the uptake ratios and autoradiographic derived % specific binding. The authors could feasibly distinguish between symptomatic and asymptomatic plaques, the detection technique had a moderate sensitivity (78%) and specificity (74%). ^11^C-PK11195 has also been evaluated as a neuroinflammation agent [91], its potential clinical use in determining early onset arthritis activity [92] and* in vivo* imaging of patients with early rheumatoid synovitis [93]. Despite its limitations with quantitative analyses, renewed attention was shown to ^18^F-DPA-714 for imaging rheumatoid arthritis, a molecule that was earlier examined in models of acute neuroinflammation, breast cancer, and glioma [94]. ^18^F-DPA-714 is a radioactive analogue of the TSPO overexpressed by macrophages; it was recently examined by French researchers in a rat model of rheumatoid arthritis employing inactive* Mycobacterium tuberculosis *[94]. The severity of the disease in the joints was evaluated from a combination of clinical symptoms, scintigraphy, histology, and immunohistochemistry. Peak inflammation after 20 days was visible in the swollen ankles, an observation correlating with higher radiotracer uptake and a T : B ratio of 3 (inflamed rats versus normal rats), enhanced TSPO expression in the hind paws, and radioactivity mainly associated with the infiltrated macrophage cells. These authors suggested ^18^F-DPA-714 may be useful to determine the disease activity during its early stages, with high potential in the clinic for imaging peripheral inflammation. The same research group also found ^18^F-DPA-714 was suitable for studying IBD in two rat models (dextran sodium sulfate (DSS) = global colonic inflammation, TNBS = local acute inflammation) [95]. Eight days after the inflammation was initiated, from the 60 min dynamic images were derived T : B ratios of 3 (DSS) and 3 (TNBS) that were not significantly different to ^18^FDG (at 7 days). The PET signal was correlated with increased TSPO expression at the cellular level, and it was stated that the ^18^F-DPA-714 probe could evaluate the level and localization of a colonic inflammation.

*Liposomes.* A liposome is a unilamellar vesicle composed of one lipid bilayer of amphipathic molecules enclosing an aqueous compartment. The aqueous compartment can be used to encapsulate an active drug, and the outer surface layer is modified (i.e., polyethylene glycol coated) to reduce immunogenicity or antigenicity. These “stealth” liposomes have strongly reduced uptake by the mononuclear phagocyte system and prolonged uptake in circulating blood. Liposomes are particles with diameters in the broad range of 50–250 nm [96], and these have been employed in diagnostic imaging modalities such as MRI (membrane-attached gadolinium ions), nuclear medicine (membrane-attached ^99m^Tc, ^111^In, or ^186^Re), CT (membrane-attached iodinated contrast agents), and sonography (encapsulated carbon dioxide) [97]. HYNIC-liposomes were prepared using a PEG 2000 derivative of distearoyl phosphatidyl-ethanolamine (dSPEA), partially hydrogenated egg-phosphtidylcholine, cholesterol, and the HYNIC-derivative of dSPEA [98]. Particles were measured as 80–85 nm diameter and these were ^99m^Tc-labeled with >95% efficiency in the presence of tricine, stannous ions, and ^99m^Tc-pertechnetate at room temperature. In a rat cecal ligation and puncture model that creates an abdominal abscess, ^99m^Tc-HYNIC-PEG-liposomes were evaluated against ^99m^Tc-HYNIC-hIgG and ^67^Ga-citrate [98]. ^99m^Tc-HYNIC-PEG-liposomes clearly showed the abscesses on the images at 2 hours pi (versus 4 hours for the other radiotracers), but only ^99m^Tc-HYNIC-PEG-liposomes and ^67^Ga-citrate correlated well with the extent of inflammation by histological evidence. After 24 hours pi, the percentage of the injected dose at the infection site was 3% ^99m^Tc-HYNIC-PEG-liposomes, 2% ^99m^Tc-HYNIC-hIgG, and 2% ^67^Ga-citrate. The authors favoured radioliposomes because of early visualization of the abscess and better correlation with the disease severity. The same group investigated ^99m^Tc-HYNIC-PEG-liposomes versus ^99m^Tc-HMPAO-PEG-liposomes in a rat model of* S. aureus* muscle infection [99]. ^99m^Tc-HMPAO-PEG-liposomes were prepared in 70–80% efficiency, and the unbound ^99m^Tc-HMPAO was removed using a size exclusion column in an extra purification step. At 2, 8, and 24 hours pi, the respective T : B ratios were (i) 14, 26, and 43 for ^99m^Tc-HYNIC-PEG-liposomes and (ii) 12, 19, and 37 for ^99m^Tc-HMPAO-PEG-liposomes. The latter radiotracer showed less* in vivo* stability and more nontarget organ uptake, in contrast to the authors' preferred HYNIC-conjugated liposome formulation. Further investigation of ^99m^Tc-labeled liposomes in a clinical study of patients with Crohn's disease found a moderate relationship between radiotracer uptake and the disease activity index, but it was prematurely terminated because of unacceptable side effects such as dyspnea and facial erythema in 3 of 9 patients [100]. A review of the literature has revealed a number of liposome formulations exist that encapsulate pharmaceuticals for treating cancer (i.e., doxorubicin, lurtotecan) or fungal infections (i.e., amphotericin B) [97]. Commercially available amphotericin formulations Abelcet and AmBisome have considerable information under “undesirable effects/adverse reactions” in their accompanying Product Information Sheets [101, 102], perhaps coinciding with a renewed interest by regulatory agencies toward the registration of liposome-containing products [103, 104]. In terms of radioliposomes for diagnostic imaging, the research activities currently belong in the preclinical domain [105, 106].

*Interleukins.* Interleukins (IL) are a group of cytokines mainly synthesized by helper CD4 T lymphocytes, monocytes, macrophages, and endothelial cells. Cytokines are secreted proteins and/or signalling molecules that promote the development and differentiation of T, B, and hematopoietic cells, as well as support the immune system function. At least 40 different interleukins have already been identified so far, and for this chapter only the radiolabeled interleukins-1, -2, -8 and -12 are described because of their relevance in detecting infection/inflammation. 

*Interleukin-1*. The IL-1 family is associated with acute and chronic inflammation, and it plays a typically proinflammatory role in the nonspecific innate response to infection. The subtype IL-1*α* is synthesized intracellularly by primary cells (i.e., keratinocytes, thymic epithelium, hepatocytes, endothelial cells, fibroblasts, epithelial cells of mucous membranes); the unprocessed precursor and processed protein are released after cell death when both actively bind to the IL-1R receptor, or for monocytes and B-lymphocytes, membrane-bound-IL-1*α* is also active [107]. At an inflammatory site, necrotic cells release IL-1*α* precursor which binds to the IL-1R receptor on nearby tissue macrophages and epithelial cells. This event initiates a proinflammatory response when a sudden influx of recruited neutrophils and then monocytes access the site. Radioiodinated (^125^I, ^131^I, and ^123^I) IL-1*α* was prepared via the iodogen method and then examined in a mouse model of* S. aureus* muscle infection against the nonspecific control protein ^131^I-myoglobin [108]. ^123^I-IL-1*α* resulted in maximal uptake in the abscess after 12 hours, a much higher value than ^131^I-myoglobin. The T : B ratios at 48 hours pi were 44 for ^123^I-IL-1*α* and 6 for ^131^I-myoglobin. The authors stated that a specific retention mechanism occurred at the focal infection, from a presumed interaction between ^123^I-IL-1*α* and IL-1R on local leukocytes.

The subtype IL-1*β* is also synthesized intracellularly by monocytes, macrophages, dendritic cells, B-lymphocytes, and natural killer (NK) lymphocytes. The inactive precursor molecule is converted into the active cytokine with the cysteine protease caspase-1 that also requires activation with ASC, NRLP3, or other trigger molecules, by noncaspases such as proteinase 3, elastase, matrix metalloprotease 9, or granzyme A extracellularly, and by autophagy [107]. At an inflammatory site, IL-1*β* enhances T-cell activation and recognition of the antigen, by acting in conjunction with other interleukins to develop or differentiate T- helper 17 cells that subsequently release IFN*γ* and prostaglandins amid the leukocyte* milieu*. IL-1ra is a receptor antagonist protein that binds with high affinity to IL-1R, IL-1*α*, and IL-1*β* causing inhibition and consequently IL-1 signalling is reduced in the inflammatory cascade. The potent inhibitory effect of IL-1ra was investigated for treating the autoinflammatory diseases Muckle-Wells and gout [109]. ^123^I-IL-1*β* was prepared via the Bolton-Hunter method (25–35% efficiency), and then the purified radiotracer was examined in rabbits with* E. coli* muscle infections against ^123^I-IL-1*α*, ^123^I-IL-1ra, and ^123^I-fMLFK [110]. The receptor binding affinity for the radioiodinated molecules was preserved, 50–75% in the presence of excess cell receptors. All four agents localized in the infectious foci from 4 hours pi onwards, the T : B ratio of ^123^I-IL-1*β* peaked at 8 hours pi, and at 20 hours pi the T : B ratios were 7 for ^123^I-IL-1*β*, 4 for ^123^I-IL-1*α*, 4 for ^123^I-IL-1ra, and 3 for ^123^I-fMLFK. Unfortunately side effects such as hypotension and headache were observed during a clinical trial, due to the biological activity of IL-1 even at very low doses of 10 ng/kg, and this precluded further application of radiolabeled IL-1 [111]. The authors initially favoured ^123^I-IL-1*β* because of its higher uptake early, plus a higher T : B ratio than the other radiotracers, but they also noted ^123^I-IL-1ra may be important in human studies because it promised fewer side effects. IL-1ra was also radiolabeled with ^18^F-fluoroacetaldehyde [112], however it has only been investigated in normal rats thus far [113]. More recently, a particularly interesting dual-domain cytokine containing IL-1ra and IL-18 binding protein was labeled with ^99m^Tc for specific inflammation targeting [114]. Interleukin-18 is another proinflammatory cytokine that induces production of TNF*α* and IL-1*β* in mononuclear cells. IL-18 bp is a secreted extracellular protein that acts as a decoy receptor; it binds and then deactivates IL-18. Large scale bioengineering techniques were used to prepare the dual-derivatized Fc fragment IL-18 bp-Fc-IL-1ra. Reduction of disulfide bonds in the Fc fragment gave the thiolated IL-18 bp-Fc-IL-ra, which in the presence of ^99m^Tc, stannous ions and glucohepatonate gave ^99m^Tc-IL-18 bp-Fc-IL-1ra in >95% yield. Competitive binding studies showed specific targeting of this radiotracer to rat PMNs. A mouse ear edema model was used to show comparable uptake of ^99m^Tc-IL-18 bp-Fc-IL-1ra to ^111^In-neutrophils at the lesion site, the T : B ratio of ^99m^Tc-IL-18 bp-Fc-IL-1ra was 9 at 3 hours pi (or 4 when excess unlabeled IL-18 bp-Fc-IL-1ra was administered 30 minutes earlier). The authors concluded that this radiotracer had potential for detecting inflammatory sites. 

*Interleukin-2.* IL-2 or* T-cell growth factor* is an *α*-helical protein of 15 kDa produced by antigen-activated T cells that can promote T-cell-dependent immune responses. IL-2 binds on target cell membrane with high affinity to a receptor consisting of the three subunits IL-2R*α* (or CD25), IL-2R*β* (or CD122), and IL-2R*γ* (or CD132). IL-2R*β* and IL-2R*γ* are important for intracellular signal transmission, and IL-2 binding to IL-2R*α* mediates IL-2R*β* activity [115]. IL-2R *α*, *β*, and *γ* are expressed by CD4+ regulatory T cells or recently antigen-activated T cells, and IL-2R *β* and *γ* are prominent on memory CD8+ T cells and NK cells. In response to a pathogen, there is modulated IL-2R signalling and expression of IL-2/IL-2R that results in clonal expansion of T cells and then differentiation into effector T cells. After the pathogen is cleared, there is a contraction phase in which most of the T cells undergo apoptosis, and the few survivors persist as memory T cells for long-term protection of the host against the same pathogen [116]. ^99m^Tc-HYNIC-IL-2 was prepared in high yield using recombinant human IL-2 [117], a stable agent in human serum at 37°C for 6 hours, and capable of stimulating cell proliferation* in vitro *[118]. A positron emitting derivative* N*-(4-^18^F-fluorobenzoyl)-IL-2 was prepared in ~30% yield; this radiotracer was stable in plasma and also capable of stimulating T cells [119]. In this work the authors examined ^18^F-FB-IL-2 in immunodepressed mice that received a xenograft (Matrigel), in which there was migration of active T lymphocytes to the inflamed xenograft periphery because of ^18^F-activity visible on the 30 minute images. They concluded that this PET radiotracer could be useful in detecting activated lymphocytes in pathological conditions such as autoimmune diseases and graft rejection. ^99m^Tc-HYNIC-IL-2 was used to image atherosclerotic plaques in end-stage renal disease patients (ESRD) [120]; 91% of cases showed plaque uptake on the images and a T : B ratio of 2 in 0.8 mm plaques ±calcification at 1 hour pi. The authors supported ^99m^Tc-HYNIC-IL-2 as a potential diagnostic agent for inflamed and vulnerable atherosclerotic plaques within the common carotid artery in ESRD patients. ^123^I-IL-2 was prepared in the presence of lactoperoxidase/glucoperoxidase in PBS and after 30 minutes, the HPLC-purified radiotracer was examined in patients with active Crohn's disease versus healthy volunteers [121]. Intestinal uptake was higher in the active Crohn's patients and this result positively correlated with the disease activity index. The same authors used autoradiography to confirm there was specific binding of ^125^I-IL-2 to IL-2R positive mononuclear cells infiltrating the gut wall (obtained from surgical specimens). 

*Interleukin-8.* IL-8 or* CXCL8* is a 72 amino acid protein of 8 kDa produced by leukocytes, an important molecule for innate immunity because of its effects on neutrophil chemotaxis and activation [122]. The inactive precursor protein (99 amino acids) is synthesized intracellularly, followed by a cleavage reaction at the N-terminus to produce a 77-amino-acid protein in fibroblasts and endothelial cells, or a 72-amino-acid protein in leukocyte cells. The 77-amino-acid isoform is secreted from nonimmune cells and then cleaved into the active 72-amino-acid CXCL8 protein. CXCL8 exists in monomer or dimer forms that are both capable of activating and regulating its two cell surface receptors [122]. It is a proinflammatory chemokine belonging to one of two main types with the acronym CXC rather than CC (or two minor types C and CX_3_C). The structural moiety of CXC is characterized as two N-terminal cysteines separated by one amino acid; for CC there are two N-terminal cysteines adjacent to each other [123]. CXCL8 mediates its effects via binding to 2 heterotrimeric G protein-coupled receptors CXCR1 and CXCR2, found on neutrophils, monocytes, macrophages, basophils, T-lymphocytes, and endothelial cells. When CXCR1 and CXCR2 are expressed, a cascade of signalling follows, as local cells adapt to the stressed or inflamed environment. ^99m^Tc-HYNIC-IL-8 has been extensively investigated in the preclinical domain for scintigraphic detection of infection and inflammation. In rabbits with intramuscular* E. coli* abscesses, ^99m^Tc-HYNIC-IL-8 resulted in a T : B ratio of 127 at 8 hours pi versus the control molecule ^99m^Tc-HYNIC-lysozyme with a ratio of 7 at the same time point [124]. The authors also noted a mild transient drop of leukocyte counts. In rabbits with TNBS-induced colitis, ^99m^Tc-HYNIC-IL-8 resulted in a T : B ratio of 18 at 4 hours pi versus ^99m^Tc-HMPAO-granulocytes with a ratio of 4 at the same time [125]. With an increasing severity of the colonic insult as normal, inflamed, and ulcerated, the respective T : B ratios at 4 hours were 4, 13, and 26 for ^99m^Tc-HYNIC-IL-8 and 1, 3, and 6 for ^99m^Tc-HMPAO-granulocytes. A rabbit model of acute pyogenic osteomyelitis (sodium morrhuate and* S. aureus *were inserted into the medullary cavity of the right femur) was employed to examine ^99m^Tc-HYNIC-IL-8 against ^111^In-gramulocytes, ^67^Ga-citrate, and ^99m^Tc-MDP [126]. In this model the T : B ratios at 4 hours pi were 6 for ^99m^Tc-HYNIC-IL-8, 1 for ^111^In-granulocytes, 2 for ^99m^Tc-MDP, and 1 for ^67^Ga-citrate. The superior T : B ratio for ^99m^Tc-HYNIC-IL-8 over the other radiotracers at that time was due to a faster clearance and therefore lower background activity. This study supports the hypothesis that ^99m^Tc-HYNIC-IL-8 predominantly binds with circulating neutrophils that ultimately migrate into the inflamed tissue and extravasate at the abscess. The same research group examined ^99m^Tc-HYNIC-IL-8 as a pulmonary infection agent in three rabbit models, with immunocompromised animals exposed to* A. fumigatus*,* S. pneumonia,* and* E. coli* [127]. The T : B ratios of ^99m^Tc-HYNIC-IL-8 at 6 hours pi were 3 for the aspergillosis model, 3 for the* S. pneumoniae* model, and 4 for the* E. coli* model. It was surmised that this radiotracer showed excellent visualization of a pulmonary infection from all three models simulating immunocompromised conditions. A more recent clinical investigation of ^99m^Tc-HYNIC-IL-8 was reported in 20x patients with suspected infections (9x suspected joint prosthesis infections, 8x osteomyelitis, 1x liver abscess, 2x soft-tissue infections) [128]. ^99m^Tc-HYNIC-IL-8 localized the infection in 10/12 patients at 4 hours pi, presenting 2x false negatives (1x vertebral osteomyelitis, 1x infected knee prosthesis), and 8/8 patients with noninfectious disorders gave no radiotracer uptake. No side effects were exhibited by patients in the study, the high efficacy was coupled with early imaging, and the authors suggested ^99m^Tc-HYNIC-IL-8 was a promising new tool for detecting infections. Another noted radiotracer includes the CXCR2-specific chemokine ^99m^Tc-HYNIC-NAP-2 (neutrophil activating peptide-2) that was investigated in a rabbit model of intramuscular* E. coli* infection, and it yielded a T : B ratio of 80 at 6 hours pi [129]. 

*Interleukin-12*. IL-12 is a 70 kDa cytokine composed of four *α*-helices, but it is described as a* heterodimer* because of two similar subunits (IL-12p35, IL-12p40) covalently linked in the structure [130]. Each subunit is expressed from different chromosomes. IL-12p40 is produced predominantly by activated monocytes, macrophages, neutrophils, and dendritic cells. IL-12p40 has been shown to act as a chemoattractant for macrophages and promotes the migration of stimulated dendritic cells. The p40 subunit is associated with several pathogenic inflammatory responses such as silicosis, graft rejection, and asthma, but it is also found to be protective in a mycobacterial infection model [131]. IL-12 is important in bridging early nonspecific innate resistance and the subsequent antigen-specific adaptive immunity. IL-12 activity is mediated via binding to a membrane receptor complex composed of two subunits IL-12R *β*1 and IL-12R *β*2. IL-12R *β*1 binds with high affinity the IL-12p40 subunit; an activation of Janus kinases leads to the phosphorylation of the receptor which becomes a docking site for STAT4 proteins, and then bound protein is phosphorylated by Janus kinases. IL-12R *β*2 recognizes either the heterodimer IL-12 or the IL-12p35 subunit and is expressed at low levels after T-cell receptor stimulation. Expression of this receptor subunit is critically influenced by IL-12 and interferon-*γ* [131]. ^99m^Tc-HYNIC-IL-12 was prepared in 75–85% efficiency and then required a purification step before further use. The radiotracer was assayed to have high specific binding to KIT225 cells that express the IL-12 receptor, and an examination in mice with TNBS-induced colitis resulted in a significant accumulation at the inflamed site versus negligible colon uptake in normal mice [132]. 

*Smaller Molecules*. C5a is a 74-amino-acid peptide with a four-helix core structure that participates in the complement cascade in innate immunity. This peptide binds to two receptors C5a1 and C5a2 typically expressed by neutrophils, macrophages, and cells of myeloid origin [133]. C5a induces smooth muscle contraction, increases vascular permeability, and induces expression of adhesion molecules on endothelial cells [134]. The C5a receptor is expressed on PMNs, it has been characterized using ^125^I-C5a [135], and there was 40% binding of ^99m^Tc-HYNIC-C5a to it on granulocytes via an* in vitro* assay [136]. ^99m^Tc-HYNIC-C5a and its natural metabolite ^99m^Tc-HYNIC-C5adR were examined in rabbits with* E. coli* muscle infections, resulting in T : B ratios of 76 and 14, respectively, at 5 hours pi [136]. The authors found the less potent C5adR analogue to be inadequate for infection/inflammation imaging, while the very potent C5a analogue was also unsuitable at the clinical level because of its risky side effects [134].

Human neutrophil peptide- (HNP-) 1 belongs to the family of defensins that kill bacteria by electrostatic interference at the cell membrane, thereby causing increased permeability [137]. HNP-1 is stored in the granules of human neutrophils and it contributes to killing bacteria during phagocytosis. ^99m^Tc-HNP-1 was radiolabeled directly, and then evaluated in mice with muscle infections (*S. aureus*,* K. pneumoniae*) against ^99m^Tc-IgG [137]. At 1 hour pi for* S. aureus* and* K. pneumoniae* models, respectively, ^99m^Tc-HNP-1 gave T : B ratios of 2 and 2, versus ^99m^Tc-IgG ratios of 2 and 1. Over the next three hours the T : B ratios for the ^99m^Tc-HNP-1 decreased, in contrast to those that increased for immunoglobulin. The same researchers compared the ^99m^Tc-HNP-1 against antimicrobial peptide ^99m^Tc-UBI 29–41 (plus other ubiquicidin fragments) in mice with muscle infections (*S. aureus*,* K. pneumoniae*). At 60 minutes pi ^99m^Tc-UBI 29–41 in* S. aureus* and* K. pneumoniae* gave T : B ratios of 2 and 2, respectively, it was preferred by the authors over ^99m^Tc-HNP-1 because of easier preparation and lack of immunological effects [138]. In the same study ^99m^Tc-UBI 29–41 gave a T : B ratio of 1 in a LPS-induced inflammation in mice, evidence of the radiotracer specifically binding to bacteria at an infectious site. ^99m^Tc-UBI 29–41 was also examined in mouse models of thigh muscle infections (*S. aureus*,* K. pneumoniae*,* C. albicans*) or LPS-induced inflammation to yield T : B ratios of 4, 2, 3, or 1, respectively, at 2 hours pi, further supporting preferential binding to microbes rather than inflammatory cells at the lesion site [139]. The authors also showed that ^99m^Tc-ciprofloxacin does not significantly distinguish between infection and inflammation in their rodent models. Direct labeling with ^99m^Tc was suggested to occur via Tc(V)=O coordinated with lysine and arginine [140]; however, the original researchers pursued an instant kit version using HYNIC-UBI 29–41 and stannous chloride starting materials [141]. ^99m^Tc-HYNIC-UBI 29–41 was prepared in >96% yield without the need of a purification step; it was stable for 24 hours and had 40% binding affinity to multidrug-resistant* S. aureus in vitro*. In the same study, this radiotracer gave a T : B ratio of 2 (versus 3 for ^99m^Tc-UBI 29–41) at 60 minutes pi in* S. aureus* infected mouse thigh muscle. ^99m^Tc-HYNIC-UBI 29–41 was renally excreted faster than ^99m^Tc-UBI 29–41. In a small clinical trial of 18 patients with suspected bone, soft tissue or prosthesis infections, ^99m^Tc-UBI 29–41 gave mean T : B ratios of 3 at 30 minutes then 2 at 120 minutes pi [142]. The diagnostic value was rated as 100% sensitivity, 80% specificity, and 94% accuracy, and no adverse effects were noted up to 4 days later. The authors concluded that ^99m^Tc-UBI 29–41 was a promising infection imaging agent, with optimal visualization at 30 minutes pi.

#### 2.4.4. Summary

In summary, the medical and scientific literature provides ample evidence of researchers driving their activities toward designing smaller radiotracer molecules with higher specificity for cells or other endogenous molecules, rather than large macromolecules that can have undesirable side effects. Peptides generally have better extraction from the circulation and higher uptake at the target site than macromolecules, but they can also invoke adverse reactions [143], even with a potency below the nanomole level [144, 145]: important precautionary information for any scientist undertaking radiotracer design. Nuclear medicine clinics are still awaiting the optimal scintigraphic imaging agents capable of discriminating between an infection and inflammation, and between fungal and bacterial infections [134, 143, 146]. The techniques to prepare radiotracers are becoming more efficient and involve simpler operations, although the PET radiotracers that promise better images are still in their infancy. Even if a radiotracer does not succeed as a clinical investigational agent, the experimental journey nevertheless creates knowledge and a better understanding of the immune response to an infection or inflammatory event.

## Figures and Tables

**Table 1:** Physical characteristics of parent radioisotopes.

Parent isotope	Origin	Reaction from grandparent	Half-life	Decay reaction to daughter	Mode of decay	Useful energy (keV)	Abundance (%)	Supplied form
^ 99m^Tc	Reactor	^ 235^U(n,f)^99^Mo ^99^Mo →^99m^Tc (*β*)	6.0 hr	^ 99m^Tc →^99^Tc	Isomeric transition, *γ*	140	86	Generator^a^

^ 111^In	Cyclotron	^ 112^Cd(p,2n)^111^In	2.8 day	^ 111^In (*β* ^−^) →^111^Cd (*γ*)	Electron capture, *γ*	172, 247	91, 94	Solution

^ 67^Ga	Cyclotron	^ 68^Zn(p,2n)^67^Ga	78.3 hr	^ 67^Ga →^67^Zn (*γ*)	Electron capture, *γ*	93, 185, 300, 393	38, 21, 17, 5	Solution

^ 18^F	Cyclotron	^ 18^O(p,n)^18^F	109.8 min	^ 18^F →^18^O (*β* ^+^)	Positron emission	average 249 (max⁡633)	97	Solution
				^ 18^F →^18^O (*γ*)	Electron capture, *γ*	511	3	

^a^Grandparent isotope is localized in the stationary phase of a column, and the parent isotope is isolated by elution with a mobile phase.

**Table 2:** Commercial radiopharmaceuticals currently used in clinical nuclear medicine.

Product trade Name/code	Manufacturer/sponsor		Final dose preparation^a,b^	Mode of administration	Indications^c^
	Origin	Description			
CERETEC	UK	Kit for the preparation of technetium [^99m^Tc] exametazime injection	Simple	Intravenous	Scintigraphic localization of seizure foci in patients with temporal lobe epilepsy

LEUCOCYTE Labelling Kit [LLK]	Australia	Kit for the preparation of technetium [^99m^Tc] labelled leucocytes (^99m^Tc-Sn(O)F_2_)	Complex	Intravenous	Image acute inflammation or infection

MDP	Many	Kit for the preparation of technetium [^99m^Tc] medronate injection	Simple	Intravenous	Skeletal imaging pharmaceutical

Indium [^111^In] oxine	UK, Netherlands	A diagnostic radiopharmaceutical intended for radiolabelling	Simple	Intravenous	*In vitro* radiolabeling of separated leukocytes and platelets which are subsequently reinjected intravenously for investigative purposes

Gallium [^67^Ga] citrate injection	Many	An injection containing radioactive gallium [^67^Ga]	Simple	Intravenous	It may be useful in demonstrating the presence and extent of Hodgkin's disease, lymphomas, and bronchogenic carcinoma and may also be useful as an aid in detecting some acute inflammatory lesions

Fluorodeoxyglucose [^18^F] injection	Many	A positron emitting radiopharmaceutical containing no-carrier added radioactive 2-deoxy-2-[^18^F]fluoro-D-glucose	Simple	Intravenous	Identification of regions of abnormal glucose metabolism associated with foci of epileptic seizures, in the evaluation of malignancy in patients and identification of left ventricular myocardium with residual glucose metabolism and reversible loss of systolic function

LeukoScan	USA, Germany	Kit for the preparation of ^99m^Tc-labeled LeukoScan	Simple	Intravenous	For determining the location and extent of infection/inflammation in bone in patients with suspected osteomyelitis, including patients with diabetic foot ulcers

Scintimun	France	Scintimun 1 mg kit for radiopharmaceutical preparation	Complex	Intravenous	For determining the location of inflammation/infection in peripheral bone in adults with suspected osteomyelitis

Nanocoll	Italy	Kit for radiopharmaceutical preparation (^99m^Tc-HAA)	Simple	Intravenous	Bone marrow scanning (excluding hematopoietic activity) and inflammation scanning (excluding the abdomen)

^a^Simple operations involving one radioisotope reconstitution step/withdrawal of final patient dose(s); ^b^complex operations involving multisteps of reconstitution/heating/other manipulations/purification/withdrawal of final patient dose(s); ^c^from the Product Information Sheet.

**Table 3:** Key parameters of radiolabeling methods.

Radiopharmaceutical		Radiosynthesis parameters			Potential burden to operator	
Simple operation	Complex operation	Process time (hr)	±blood cells	Special equipment	Radiation^*^	Biological
^ 99m^Tc-HMPAO	—	<0.5	−	—	Low	−
^ 99m^Tc-MDP	—	<0.2	−	—	Low	−
^ 99m^Tc-sulesomab	—	<0.2	−	—	Low	−
^ 99m^Tc-albumin nanocolloid	—	0.5	−	—	Low	−
^ 111^In-oxine	—	0	−	—	Medium	−
^ 67^Ga-citrate	—	0	−	Thicker Pb pots	Medium	−
^ 18^FDG (supplied)	—	0	−	Thicker Pb pots/dispensing pig	High	−
—	^ 99m^Tc-tin fluoride colloid	<0.9	−	rotation mixer	Low	−
—	^ 99m^Tc-SnF_2_ colloid-leukocytes	1	+	Rotation mixer, centrifuge	Low	+
—	^ 99m^Tc-HMPAO-leukocytes	<1.5	+	Centrifuge	Low	+
—	^ 111^In-oxine-leukocytes	<1.5	+	Centrifuge	Medium	+
—	^ 99m^Tc-besilesomab	<0.2	−	—	Low	−
—	^ 18^FDG (on-site)	~2.5	−	Cyclotron, hot cells, synthesis modules, (autoclave), etc	High	−

^*^Broadly based on the energy of emissions and number of emissions: low = ^99m^Tc; medium = ^111^In, ^67^Ga; high = ^18^F.

**Table 4:** Characteristics of selected radiotracers.

Radiotracer molecules			Dose formulation	Radiolabeling		Radiotracer purification method
Isotope	Isotope chelator	Cold moiety		Conditions	Efficiency	
Synthetics						
^ 111^In	DTPA	fMLFK	Sephadex G-10 eluent	RT/30 min	95%	RP-HPLC
^ 99m^Tc	HYNIC	fMLFK	Sodium phosphate, saline, pH 8	RT/30–60 min	>96%	RP-HPLC
^ 99m^Tc	HYNIC	MLF-OMe	Sodium phosphate, saline, pH 8	RT/30–60 min	>96%	RP-HPLC
^ 68^Ga	None	Citrate	Sodium citrate, pH 6-7	90°C/10 min	>98%	Anion exchange column
^ 68^Ga	None	TAFC	1.1 M sodium acetate, pH 6-7	RT/15 min	≥95%	—
^ 68^Ga	None	FOXE	1.1 M sodium acetate, pH 6-7	80°C/20 min	≥95%	—
^ 111^In	DTPA	LTB-4 (DPC11870-11)	Ammonium acetate, pH 5.5	RT/30 min	95%	RP-HPLC
^ 99m^Tc	HYNIC	LTB-4 (MB88)	Tricine, isonicotinic acid, PBS, SnSO_4_, pH 7	100°C/30 min	95%	RP-HPLC
^ 18^F	pFBA	LTB-4 (MB67)	Sodium acetate, PBS, pH 4–7	50°C/>30 min	65–85%	Evaporation, Δ & N_2_↑

Antibiotics						
^ 99m^Tc	None	Fluconazole	PYP, SnCl_2_, KBH_4_ in 0.1 M NaOH, saline, PBS, pH 7.5	RT/120 min	90%	—
^ 99m^Tc	None	Ciprofloxacin	Sn(II) tartrate	RT/15 min	>40%	—
^ 99m^Tc	None	Enrofloxacin	Ethanol : water [1 : 1]	60°C/10 min	81%	SepPak tC2 cartridge
^ 99m^Tc	None	Pefloxacin	SnCl_2_, pH 6	100°C/30 min	98%	—
^ 99m^Tc	None	Ceftizoxime	Sodium dithionite, N_2_↑	100°C/5 min	>90%	—
^ 99m^Tc	None	Vancomycin	SnCl_2_, pH 6-7	RT/10 min	99%	—
^ 99m^Tc	None	Kanamycin	SnCl_2_, pH 6-7	RT/25–30 min	99%	—
^ 99m^Tc	None	Alafosfalin	SnCl_2_, pH 6	RT/5–10 min	90–95%	—
^ 99m^Tc	None	Phosphomycin	SnCl_2_, pH 7	RT/<1 min	98%	—
^ 99m^Tc	None	Cefoperazone	SnCl_2_, pH 8	RT/10 min	98%	—
^ 99m^Tc	None	Cefuroxime axetil	SnCl_2_, pH 5	RT/15 min	92%	—
^ 99m^Tc	[^99m^Tc(CO)_5_]	Novobiocin	Water	RT/30 min	99%	—

Biological origin						
*Cells: *						
^ 18^FDG	None	Leukocytes	Autologous LEuk, saline	37°C/30 min	79% ^18^F-gran.	Centrifugation
^ 111^In	Oxine	Dendritic cells	Allogeneic DC, saline	RT/60 min	94%	Centrifugation
^ 111^In	Oxine	Lymphocytes	Autologous LYM, lym poor plasma	37°C/10 min	77%	Centrifugation
*Macromolecules *						
^ 111^In	DTPA	hpc-IgG	Citrate	RT/instant	>95%	Centrifugation
^ 99m^Tc	None	hpc-IgG	Tartrate, SnCl_2_	RT/20 min	90%	—
^ 99m^Tc	HYNIC	hpc-IgG	Acetate, tricine, SnSO_4_	RT/15 min	>95%	—
^ 99m^Tc	None	Rat pc-IgG	MDP, SnCl_2_, PBS, N_2_↑	RT/20 min	≥94%	—
^ 99m^Tc	None	HSA	Saline, SnCl_2_, N_2_↑, pH 2–7	RT/20 min	≥95%	—
^ 99m^Tc	None	Infliximab	MDP, SnCl_2_, PBS, N_2_↑	RT/20 min	≥91%	—
^ 99m^Tc	None	Fanolesomab	Maltose, tartrate, succinic acid, Sn(II) tartrate, glycine, EDTA, ascorbic acid, pH 6	37°C/30 min	≥90%	—
^ 99m^Tc	HYNIC	E-selectin peptide (IMP-178)	2-OH-*β*-cyclodextrin, Sncl_2_, acetate, ascorbate	RT/10 min; 100°C/15 min	92%	—
^ 123^I	None	B2702-p (75–84)	PBS, pH 7	RT/15 min	>80%	Anion exchange column
^ 99m^Tc	[^99m^Tc(CO)_5_]	B2702-p (+his-gly)	PBS, pH 7	100°C/15 min then 80°C/20 min	>90%	—
^ 99m^Tc	HMPAO	PEG-liposomes	5% glucose	RT/15 min	70–80%	Size exclusion on PD-10 column
^ 123^I	None	hr-IL-1*α*	0.5% BSA in PBS	RT/10 min	50–80%	Sephadex G-25 column/0.2 *μ*m filter
^ 123^I	None	hr-IL-1*β*	0.25% gelatine in PBS	RT/10 min; ice/30 min	25–35%	Sephadex G-25 column/0.2 *μ*m filter
^ 18^F	[para-^18^F-fluoroethyl]	hr-IL-1ra	PBS	38°C/45 min	7–24%	HiTrap column/filter
^ 99m^Tc	None	IL-18bp-Fc-IL-1ra	PBS, pH 7	RT/30 min	>75%	Sephadex G-25 column
^ 99m^Tc	HYNIC	hr-IL-2	SnCl_2_, tricine, nicotinic acid, pH 6-7	RT/<60 min	>99%	—
^ 123^I	None	hr-IL-2	5% glucose	RT/30 min	>96%	HPLC; 30°C/20 min/N_2_↑
^ 18^F	pFBA	hr-IL-2	Saline, <10% ethanol	borate pH 8.5 : EtOH [1 : 1], 50°C/10 min	25–35%	RP-HPLC
^ 99m^Tc	HYNIC	hr-IL-8	SnSO_4_, tricine, nicotinic acid, 0.5% BSA in PBS, pH 7	70°C/30 min	>98%	—
^ 99m^Tc	HYNIC	hr-IL-12	Stannous tartrate, tricine, pH 6-7	RT/30 min	75–85%	G-50 microspin column
^ 99m^Tc	HYNIC	C5a	SnSO_4_, tricine, 0.5% BSA in PBS	RT/30 min	>95%	Sephadex G-25 column/0.2 *μ*m filter
^ 99m^Tc		C5adR				
*Smaller fragments: *						
^ 99m^Tc	None	UBI-29-41	Sn(II) PYP, potassium borohydride, NaOH, pH 5-6	RT/60 min	95%	—
^ 99m^Tc	HYNIC	UBI-29-41	SnCl_2_, tricine, mannitol, pH 4	RT/>60 min	>96%	—

RT: room temperature; RP: reverse phase; HPLC: high pressure liquid chromatography; none: isotope is directly bound to the structure of the nonradioactive component; pFBA: para-^18^F-fluorobenzaldehydel; Δ: heat; PBS: phosphate buffered saline; UBI: ubiquicidin; hpc: human polyclonal; IL: interleukin; hr: human recombinant; LT: leukotriene; DC: dendritic cells; LEuk: Leukocytes; LYM: Lymphocytes.

**Table 5:** Applications of selected radiotracers.

Radiotracer molecules	*In vitro* experiments	Type of model/animal		Human use	Uptake mechanism *in vivo *	Application as a potential nuclear diagnostic agent
		Infection	Inflammation			
Synthetics						
^ 111^In-fMLFK	fMLF BA, superoxide release by PMN	*S. aureus/*rats	−	?		−
^ 99m^Tc-fMLFK		*E. coli*, *S. aureus/*rabbits	Zymosan/rabbit	?	Chemotactic peptide binds to PMN & monocytes	−
^ 99m^Tc-MLF-OMe				?		−
^ 68^Ga-citrate	—	—	−	+	^ 68^Ga-transferrin forms in circulation, binds to lactoferrin in LEuK or ferritin/siderophores in pathogens	100% sensitivity, 76% specificity in bone infections
^ 68^Ga-TAFC & ^68^Ga-FOXE	Fungal BA, *in vivo* binding	*Aspergillus fumigatus/*rats	−	−	Uptake by pathogens via Fe transporter system	*A. fumigatus *(fungus) specific
^ 111^In-LTB-4 (DPC11870-11)	Receptor BA	*E. coli*/rabbits	TNBS colitis/rabbit	?	LT INF mediator participates in lipoxygenase pathway & binds to PMN after migration from bone marrow to abscess	+
^ 99m^Tc-LTB-4 (MB88) & ^18^F-LTB-4 (MB67)	—		−	?		+

Antibiotics						
^ 99m^Tc-fluconazole	Microbe/leukocyte BAs	*C. albicans*, *A. fumigatus*/mice	−	?	↓ fungal cytochrome P450 enzyme 14*α*-demethylase	*Fungal* specific
^ 99m^Tc-ciprofloxacin	Bacteria BA	*C. albicans*, *S. aureus/*rats	Turpentine/rats	+	Binding bacterial DNA gyrase	94% sensitivity, 83% specificity in orthopedic infections
^ 99m^Tc-enrofloxacin				?		+
^ 99m^Tc-pefloxacin	—	*S. aureus/*rats	−	?	↓ type II topoisomerase DNA gyrase	+
^ 99m^Tc-ceftizoxime	Antimicrobial assay	*E. coli*/rats	−	?	↓ peptidoglycan biosynthesis & binding to bacterial membrane	+
^ 99m^Tc-vancomycin	—	*S. aureus/*rats	Turpentine/rats	?	↓ cell wall biosynthesis in gram +ve bacteria & binding to membrane	+
^ 99m^Tc-kanamycin	Bacteria BA	*S. aureus/*rats, rabbits	−	?	↓ protein synthesis & binding to 30S subunit of prokaryotic ribosome	+
^ 99m^Tc-alafosfalin	Bacteria BA	*S. aureus/*rats, mice	TNBS colitis/rats	−	↓ peptidoglycan biosynthesis	−
^ 99m^Tc-phosphomycin	—	*E. coli*, *S. aureus/*mice	−	−	↓ bacterial cell wall biosynthesis & inactivating UDP-*N*-acetylglucosamine-3-enolpyruvyltransferase	−
^ 99m^Tc-cefoperazone	—	*S. aureus/*rats	Turpentine/rats	?	↓ cell wall biosynthesis	+
^ 99m^Tc-cefuroxime axetil	—	*S. aureus/*rats	Turpentine/rats	?	↓ cell wall biosynthesis & binding to essential target proteins	+
^ 99m^Tc-novobiocin	—	*S. aureus/*rats	−	−	↓ bacterial DNA gyrase & targeting enzyme GyrB subunit	?

Biological origin						
*Cells: *						
^ 18^FDG-leukocytes	Radiolabeling & purification	*P. aeruginosa, E. coli*/rats	Turpentine/rats	+	Participates in INF response	84% accuracy, in suspected infection
^ 111^In-lymphocytes		—	+	+	Participates in INF, LYM graft trapping	Diagnosis of acute kidney-graft rejection
^ 111^In-dendritic cells	DC phenotype & viability	Allogenic antigens/sheep		−	Participates in INF response, DC traffic to local lymph nodes to interact with T-LYM	−
*Macromolecules: *						
^ 111^In-DTPA-hpc-IgG	—	*E. coli, S. aureus, P. aeruginosa, K. pneumonia, C. albicans*/rats	Turpentine/rats	+	↑ VP, transchelation ^111^In to extravascular proteins	100% sensitivity, 85% specificity chronic osteomyelitis, spondylodiscitis, pulmonary INF
^ 99m^Tc-HYNIC-hpc-IgG	Histopathology	*S. aureus, P. Aspergillus*/rats	Turpentine/mice	+	↑ VP, ^99m^Tc binds to local tissue	
*Macromolecules: *						
^ 99m^Tc-hpc-IgG	—	*S. aureus/*rats	−	+	↑ VP, Fc receptor binding to local INF cells	Scintigraphic staging of IBD patients
^ 99m^Tc-rat pc-IgG			−	−		Species specific marker
^ 99m^Tc-HSA	—	*E. coli, S. aureus, P. aeruginosa, K. pneumonia, C. albicans*/rats	Turpentine/rats	−	↑ VP, no receptors for retention	Capillary permeation marker
^ 99m^Tc-infliximab	TNF*α* BAs	*S. aureus/*rats	−	+	Participating in granule release pathway, specific binding to ↑ h-TNF*α* pro-INF cytokine	In patients with refractory monoarthritis, not IBD
^ 99m^Tc-fanolesomab	—	—	−	+	Specific binding to 3-fucosyl-N-acetyl-lactosamine in CD15 antigens expressed by h-PMN & eosinophils	Withdrawn from market
^ 99m^Tc-E-Selectin peptide (IMP-178)	BAs	—	Osteomyelitis, arthritis/rat	?	Specific binding to E-selectin (CD62E) expressed on endothelium, ↑ in RA	+
^ 123^I-B2702-p (75–84) & ^99m^Tc-B2702-p (+his-gly)	Immunohistochemistry, autoradiography	—	Artherosclerotic plaques/rabbits	−	Specific binding to ↑ VCAM-1 expressed by vulnerable plaques	+
^ 99m^Tc-PEG-liposomes	Histopathology	*S. aureus, P. Aspergillus*/rats	Turpentine/mice	+	“physiologic filtration” rather than phagocytosis	Side effects [100]
^ 123^I-hr-IL-1*α* & ^123^I-hr-IL-1*β*	EL-4 BA	*S. aureus/*mice, *E. Coli*/rabbits	−	?	Participating in granule release pathway, specific binding to IL-1 receptors expressed on LEUK	?
^ 18^F-hr-IL-1ra	—	Biodistribution/normal rats		−		+
^ 99m^Tc-IL-18bp-Fc-IL-1ra	BAs	—	Ear edema/mice	?	Specific binding to IL-1 receptors expressed on PMN & IL-18	+
^ 99m^Tc-HYNIC-IL-2	CD25 BA	—	−	+	Participating in granule release pathway, specific binding to IL-2 receptors (CD25) by tissue infiltrating LYM	100% sensitivity, inflamed atherosclerotic plaque,
^ 123^I-hr-IL-2	—	—	−	+		assess influx of mononuclear cells within gut wall in CD
^ 18^F-hr-IL-2	BA	Active T-LYM in Matrigel/immune-incompetent SCID mice		?	Participating in granule release pathway & bindins to human activated T-LYM expressing CD25	Detect cell migration in autoimmune diseases, & graft rejection
^ 99m^Tc-hr-IL-8	—	*A. Fumigatus,* lung infection/rabbits	−	+	Participating in granule release pathway, specific binding to IL-8 receptors (CXCR1, CXCR2) expressed by PMN	Early detection of infections/INFs
^ 99m^Tc-HYNIC-IL-12	Receptor BA	—	Autoimmune colitis	?	Participating in granule release pathway, specific binding to IL-12 receptors on activated T or NK LYM	*In vivo* imaging of T-LYM in immune-mediated processes
^ 99m^Tc-HYNIC-C5a & ^99m^Tc-HYNIC-C5adR	Receptor BA	*E. coli*/rabbits	−	−	INF mediator in complement pathway binds to guanine nucleotide-binding inhibitory protein receptors	−
*Smaller fragments: *						
^ 99m^Tc-UBI-29-41	BA	Bacterial, *C. albicans*/mice, rabbits	Saline, LPS, dead bacteria or *C. albicans*/mice	+	Antimicrobial peptide fragment that is trapped in the cell membrane of microorganisms	100% sensitivity, 80% specificity, infective foci in bone, soft tissue
^ 99m^Tc-HYNIC-UBI-29-41	Bacteria BA	*S. aureus/*mice	−			+

PMN: polymorphonuclear neutrophils; LT: leukotriene; ?: no recent reports; h: human; DC: dendritic cells; pc: polyclonal; IL: interleukin; r: recombinant; IBD: inflammatory bowel disease; CD: Crohn's disease; LPS: lipopolysaccharide; TNBS: 2,4,6-trinitrobenzene sulfonic acid (to induce experimental colitis); INF: inflammatory; RA: rheumatoid arthritis; LEuK: leukocytes; VP: vascular permeability; LYM: lymphocytes; BA: binding assay; ↑: increases; ↓: inhibits.

**Table 6:** Status of monoclonal antibodies in 2009 [147].

mAb species origin	Number of mAb products (fraction)					
	Of total	In market^a^	Withdrawn from market	In preclinical or clinical trials	Radiolabeled mAbs^b^	
					In	Withdrawn
Murine	36/242	7/36	8/36	21/36	3/7	7/8
Chimeric^c^	24/242	11/24	0/24	13/24	0/11	0/0
Humanized^d^	127/242	12/127	1/127	114/127	0/12	1/1
Human	55/242	2/55	2/55	51/55	0/2	1/2

^a^Achieved market authorization and commercially available; ^b^diagnostic and therapy radioisotopes; ^c^structure contains one species other than human; ^d^structure contains variable chains not human.

## References

[1] Webber D. I., Zimmer A. M., Geyer M. C., Spies S. M. (1992). Use of a single-strip chromatography system to assess the lipophilic component in technetium-99m exametazime preparations. *Journal of Nuclear Medicine Technology*.

[2] Pandos G., Penglis S., Tsopelas C. (1999). Validation of a column method for technetium-99m exametazime quality control. *Journal of Nuclear Medicine Technology*.

[3] (2009). *Product Information Sheet for CERETEC*.

[4] (2011). *British Pharmacopoeia Volume IV*.

[5] Kowalsky R. J., Perry J. R. (1987). *Radiopharmaceuticals in Nuclear Medicine Practice*.

[6] Bellen J. C., Chatterton B. E., Penglis S., Tsopelas C. (1995). Gallium-67 complexes as radioactive markers to assess gastric and colonic transit. *Journal of Nuclear Medicine*.

[7] (2006). *Product Information Sheet for INDIUM OXINE*.

[8] Roca M., de Vries E. F. J., Jamar F., Israel O., Signore A. (2010). Guidelines for the labelling of leucocytes with ^111^In-oxine. *European Journal of Nuclear Medicine and Molecular Imaging*.

[9] Edreira M. M., Colombo L. L., Perez J. H., Sajaroff E. O., De Castiglia S. G. (2001). In vivo evaluation of three different 99mTc-labelled radiopharmaceuticals for sentinel lymph node identification. *Nuclear Medicine Communications*.

[10] Tsopelas C. (2005). The radiopharmaceutical chemistry of ^99m^Tc-tin fluoride colloid-labeled-leukocytes. *The Quarterly Journal of Nuclear Medicine and Molecular Imaging*.

[11] Everett D. H. (1999). *Basic Principles of Colloid Science*.

[12] Tsopelas C. (2006). Physico-chemical characterisation of ^99m^Tc-tin fluoride colloid agent used for labelling white cells. *Journal of Labelled Compounds and Radiopharmaceuticals*.

[13] Whateley T. L., Steele G. (1985). Particle size and surface charge studies of a tin colloid radiopharmaceutical for liver scintigraphy. *European Journal of Nuclear Medicine*.

[14] Hilditch T. E., Elliott A. T., Murray T., Whateley T. L. (1986). Formation of large particles in a ^99^Tc^m^-tin colloid preparation. *Nuclear Medicine Communications*.

[15] Puncher M. R. B., Blower P. J. (1995). Labelling of leucocytes with colloidal tech netium-99m-SnF2: an investigation of the labelling process by autoradiography. *European Journal of Nuclear Medicine*.

[16] Smith E., Tsopelas C., Drew P., Bartholomeusz F. D. L. (2005). Distribution of ^99m^Tc-stannous fluoride colloid (SFC) in human blood as measured by an in vitro assay employing anti-CD15-antibody-magnetic beads. *Internal Medicine Journal*.

[17] McAfee J. G., Thakur M. L. (1976). Survey of radioactive agents for in vitro labeling of phagocytic leukocytes. II. Particles. *Journal of Nuclear Medicine*.

[18] Tsopelas C., Smith E., Drew P. A., Bartholomeusz F. D. L. (2003). Preparation and biological evaluation of ^99^mTc-stannous fluoride colloid-labelled-leucocytes in rats ^99^mTc-stannous fluoride-labelled-leucocytes in rats. *Journal of Labelled Compounds and Radiopharmaceuticals*.

[19] Ramsay S. C., Gallagher H., Barnes J., Maggs J., Cassidy N., Ketheesan N. (2005). ^99^mTc-Stannous Colloid (TcSnC) labelling is complement Receptor 3 (CR3) mediated and alters neutrophil priming status. *Internal Medicine Journal (Supplement)*.

[20] Salman H., Bergman M., Bessler H., Alexandrova S., Djaldetti M. (2000). Ultrastructure and phagocytic activity of rat peritoneal macrophages exposed to low temperatures *in vitro*. *Cryobiology*.

[21] Evans D. G., Evans D. J., Graham D. Y. (1992). Adherence and internalization of *Helicobacter pylori* by HEp-2 cells. *Gastroenterology*.

[22] Offen D., Gorodin S., Melamed E., Hanania J., Malik Z. (1999). Dopamine-melanin is actively phagocytized by PC12 cells and cerebellar granular cells: possible implications for the etiology of Parkinson's disease. *Neuroscience Letters*.

[23] Mock B. H., English D. (1987). Leukocyte labeling with technetium-99m tin colloids. *Journal of Nuclear Medicine*.

[24] Tsopelas C. (2004). A study of the distribution of dextran as a sedimenting agent during the ^99m^Tc-HMPAO leukocytes labeling procedure. *Hellenic Journal of Nuclear Medicine*.

[25] Skehan S. J., White J. F., Evans J. W. (2003). Mechanism of accumulation of ^99^mTc-Sulesomab in inflammation. *Journal of Nuclear Medicine*.

[26] Gallagher B. M., Fowler J. S., Gutterson N. I., MacGregor R. R., Wan C.-N., Wolf A. P. (1978). Metabolic trapping as a principle of radiopharmaceutical design: some factors responsible for the biodistribution of [18F] 2-deoxy-2-fluoro-D-glucose. *Journal of Nuclear Medicine*.

[27] Silverman M., Aganon M. A., Chinard F. P. (1970). Specificity of monosaccharide transport in dog kidney. *The American Journal of Physiology*.

[28] Lambrecht F. Y. (2011). Evaluation of ^99^mTc-labeled antibiotics for infection detection. *Annals of Nuclear Medicine*.

[29] Fischman A. J., Pike M. C., Kroon D. (1991). Imaging focal sites of bacterial infection in rats with indium-111-labeled chemotactic peptide analogs. *Journal of Nuclear Medicine*.

[30] van der Laken C. J., Boerman O. C., Oyen W. J. G. (1997). Technetium-99m-labeled chemotactic peptides in acute infection and sterile inflammation. *Journal of Nuclear Medicine*.

[31] Gupta P., Kumar A., Singla S. (1997). Modified technique for manual synthesis of ^68^Ga-citrate with high radiochemical purity. *Journal of Nuclear Medicine*.

[32] Nanni C., Errani C., Boriani L. (2010). ^68^Ga-citrate PET/CT for evaluating patients with infections of the bone: preliminary results. *Journal of Nuclear Medicine*.

[33] Luckey M., Pollack J. R., Wayne R., Ames B. N., Neilands J. B. (1972). Iron uptake in *Salmonella typhimurium*: utilization of exogenous siderochromes as iron carriers. *Journal of Bacteriology*.

[34] Petrik M., Franssen G. M., Haas H. (2012). Preclinical evaluation of two 68Ga-siderophores as potential radiopharmaceuticals for Aspergillus fumigatus infection imaging. *European Journal of Nuclear Medicine and Molecular Imaging*.

[35] Petrik M., Haas H., Laverman P. (2014). ^68^Ga-triacetylfusarinine C and ^68^Ga-ferrioxamine e for aspergillus infection imaging: uptake specificity in various microorganisms. *Molecular Imaging and Biology*.

[36] Afonso P. V., Janka-Junttila M., Lee Y. J. (2012). LTB4 is a signal-relay molecule during neutrophil chemotaxis. *Developmental Cell*.

[37] Toda A., Yokomizo T., Shimizu T. (2002). Leukotriene B4 receptors. *Prostaglandins and Other Lipid Mediators*.

[38] van Eerd J. E. M., Oyen W. J. G., Harris T. D. (2005). Scintigraphic imaging of infectious foci with an ^111^In-LTB4 antagonist is based on in vivo labeling of granulocytes. *Journal of Nuclear Medicine*.

[39] van Eerd J. E. M., Laverman P., Oyen W. J. G. (2004). Imaging of experimental colitis with a radiolabeled leukotriene B_4_ antagonist. *Journal of Nuclear Medicine*.

[40] Van Eerd J. E. M., Broekema M., Harris T. D. (2005). Imaging of infection and inflammation with an improved ^99m^Tc-labeled LTB4 antagonist. *Journal of Nuclear Medicine*.

[41] Rennen H. J. J. M., Laverman P., van Eerd J. E. M., Oyen W. J. G., Corstens F. H. M., Boerman O. C. (2007). PET imaging of infection with a HYNIC-conjugated LTB4 antagonist labeled with F-18 via hydrazone formation. *Nuclear Medicine and Biology*.

[42] Solanki K. K., Bomanji J., Siraj Q., Small M., Britton K. E. (1993). Tc-99m Infecton: a new class of radiopharmaceutical for infection imaging. *Journal of Nuclear Medicine*.

[43] Siaens R. H., Rennen H. J., Boerman O. C., Dierckx R., Slegers G. (2004). Synthesis and comparison of ^99m^Tc-enrofloxacin and ^99m^Tc-ciprofloxacin. *Journal of Nuclear Medicine*.

[44] Sonmezoglu K., Sonmezoglu M., Halac M. (2001). Usefulness of ^99m^Tc-ciprofloxacin (infecton) scan in diagnosis of chronic orthopedic infections: Comparative study with ^99m^Tc-HMPAO leukocyte scintigraphy. *Journal of Nuclear Medicine*.

[45] Motaleb M. A. (2010). Radiochemical and biological characteristics of ^99m^Tc-difloxacin and ^99m^Tc-pefloxacin for detecting sites of infection. *Journal of Labelled Compounds and Radiopharmaceuticals*.

[46] Qaiser S. S., Khan A. U., Khan M. R. (2010). Synthesis, biodistribution and evaluation of ^99m^Tc-sitafloxacin kit: a novel infection imaging agent. *Journal of Radioanalytical and Nuclear Chemistry*.

[47] Motaleb M. A. (2009). Preparation, quality control and stability of ^99m^Tc-sparafloxacin complex, a novel agent for detecting sites of infection. *Journal of Labelled Compounds and Radiopharmaceuticals*.

[48] Motaleb M. A. (2007). Preparation and biodistribution of ^99m^Tc-lomefloxacin and ^99m^Tc-ofloxacin complexes. *Journal of Radioanalytical and Nuclear Chemistry*.

[49] Chattopadhyay S., Saha Das S., Chandra S. (2010). Synthesis and evaluation of ^99m^Tc-moxifloxacin, a potential infection specific imaging agent. *Applied Radiation and Isotopes*.

[50] Diniz S. O. F., Siqueira C. F., Nelson D. L., Martin-Comin J., Cardoso V. N. (2005). Technetium-99m ceftizoxime kit preparation. *Brazilian Archives of Biology and Technology*.

[51] Motaleb M. A. (2007). Preparation of ^99m^Tc-cefoperazone complex, a novel agent for detecting sites of infection. *Journal of Radioanalytical and Nuclear Chemistry*.

[52] Yurt Lambrecht F., Yilmaz O., Unak P., Seyitoglu B., Durkan K., Baskan H. (2008). Evaluation of  ^99m^Tc-Cefuroxime axetil for imaging of inflammation. *Journal of Radioanalytical and Nuclear Chemistry*.

[53] Fazlil A., Salouti M., Ahmadi G., Mirshojaei F., Mazidi M., Heydari Z. (2012). Radiolabeling of ceftriaxone with ^99m^Tc as a targeting radiopharmaceutical for *Staphylococcus aureus* detection in mouse model. *The Iranian Journal of Medical Physics*.

[54] Penglis S., Tsopelas C., Bartholomeusz F. D. L. (2001). ^99^mTc-phosphomycin as a potential infection imaging agent. *Hellenic Journal of Nuclear Medicine*.

[55] Tsopelas C., Penglis S., Ruszkiewicz A., Bartholomeusz F. D. L. (2003). ^99m^Tc-Alafosfalin: an antibiotic peptide infection imaging agent. *Nuclear Medicine and Biology*.

[56] Tsopelas C., Penglis S., Bartholomeusz F. D. L. (2002). Comparison of ^99m^Tc-alafosfalin and ^67^Ga-citrate in a mouse model of bacterial infection. *Nuclear Medicine Review*.

[57] Tsopelas C., Penglis S., Ruszkiewcz A., Bartholomeusz F. D. L. (2003). Inflammation imaging with ^99m^Tc-alafosfalin in a rat model of colitis. *Hellenic Journal of Nuclear Medicine*.

[58] Roohi S., Mushtaq A., Malik S. A. (2005). Synthesis and biodistribution of ^99m^Tc-vancomycin in a model of bacterial infection. *Radiochimica Acta*.

[59] Roohi S., Mushtaq A., Jehangir M., Malik S. A. (2006). Synthesis, quality control and biodistribution of ^99m^Tc-Kanamycin. *Journal of Radioanalytical and Nuclear Chemistry*.

[60] Shah S. Q., Khan M. R., Khan A. U. (2011). ^99m^Tc-novobiocin: a novel radiotracer for infection imaging. *Radiochimica Acta*.

[61] Lupetti A., Welling M. M., Mazzi U., Nibbering P. H., Pauwels E. K. (2002). Technetium-99m labelled fluconazole and antimicrobial peptides for imaging of *Candida albicans* and *Aspergillus fumigatus* infections. *European Journal of Nuclear Medicine*.

[62] Forstrom L. A., Mullan B. P., Hung J. C., Lowe V. J., Thorson L. M. (2000). 18F-FDG labelling of human leukocytes. *Nuclear Medicine Communications*.

[63] Pellegrino D., Bonab A. A., Dragotakes S. C., Pitman J. T., Mariani G., Carter E. A. (2005). Inflammation and infection: imaging properties of ^18^F-FDG-labeled white blood cells versus ^18^F-FDG. *Journal of Nuclear Medicine*.

[64] Rini J. N., Palestro C. J. (2006). Imaging of infection and inflammation with ^18^F-FDG-labeled leukocytes. *Quarterly Journal of Nuclear Medicine and Molecular Imaging*.

[65] Signore A., Soroa V. E., de Vries E. F. J. (2009). Radiolabelled white blood cells or FDG for imaging of inflammation and infection?. *Quarterly Journal of Nuclear Medicine and Molecular Imaging*.

[66] Tsopelas C., Chew G., Chew J., Coates T., Russ G. R. (2006). The distribution of allogeneic ^111^In-dendritic cells in sheep. *ANZ Nuclear Medicine*.

[67] Martin-Comin J., Roca M., Griñó J. M., Paradell C., Caralps A. (1985). Clinical usefulness of ^111^In-oxine-labeled autologous lymphocytes in kidney-graft rejection. *European Journal of Nuclear Medicine*.

[68] Claessens R. A. M. J., Koenders E. B., Boerman O. C. (1995). Dissociation of indium from indium-111-labelled diethylene triamine penta-acetic acid conjugated non-specific polyclonal human immunoglobulin G in inflammatory foci. *European Journal of Nuclear Medicine*.

[69] Thakur M. L., DeFulvio J., Park C. H. (1991). Technetium-^99^m-labeled proteins for imaging inflammatory foci. *International Journal of Radiation Applications and Instrumentation.*.

[70] Tsopelas C., Penglis S., Miller D., Rischmueller M., Bartholomeusz F. D. L. (2006). Evaluation of ^99^mTc-immunoglobulins for imaging infection in the rat. *Journal of Labelled Compounds and Radiopharmaceuticals*.

[71] Arndt J. W., van der Sluys Veer A., Blok D. (1992). A prospective comparison of ^99^mTc-labeled polyclonal human immunoglobulin and ^111^In granulocytes for localization of inflammatory bowel disease. *Acta Radiologica*.

[72] Karczmarczyk U., Markiewicz A., Mikołajczak R. (2004). ^99m^Tc human IgG radiolabelled by HYNIC. Biodistribution and scintigraphy of experimentally induced inflammatory lesions in animal model. *Nuclear Medicine Review*.

[73] Dams E. T. M., Becker M. J., Oyen W. J. G. (1999). Scintigraphic imaging of bacterial and fungal infection in granulocytopenic rats. *Journal of Nuclear Medicine*.

[74] Dams E. T. M., Oyen W. J. G., Boerman O. C. (1998). Technetium-^99^m labeled to human immunoglobulin G through the nicotinyl hydrazine derivative: a clinical study. *Journal of Nuclear Medicine*.

[75] Tsopelas C., Penglis S., Ruskiewicz A., Bartholomeusz F. D. L. (2006). Scintigraphic imaging of experimental colitis with technetium-99m-infliximab in the rat. *Hellenic Journal of Nuclear Medicine*.

[76] D'Alessandria C., Malviya G., Viscido A. (2007). Use of a ^99m^Tc labeled anti-TNF*α* monoclonal antibody in Crohn's disease: in vitro and in vivo studies. *The Quarterly Journal of Nuclear Medicine and Molecular Imaging*.

[77] Conti F., Malviya G., Ceccarelli F. (2012). Role of scintigraphy with ^99^mTc-infliximab in predicting the response of intraarticular infliximab treatment in patients with refractory monoarthritis. *European Journal of Nuclear Medicine and Molecular Imaging*.

[78] http://www.snm.org/index.cfm?PageID=4655.

[79] Shanthly N., Aruva M. R., Zhang K., Mathew B., Thakur M. L. (2006). ^99m^Tc-fanolesomab: affinity, pharmacokinetics and preliminary evaluation. *Quarterly Journal of Nuclear Medicine and Molecular Imaging*.

[80] Kipper S. L., Rypins E. B., Evans D. G., Thakur M. L., Smith T. D., Rhodes B. (2000). Neutrophil-specific ^99m^Tc-labeled anti-CD 15 monoclonal antibody imaging for diagnosis of equivocal appendicitis. *Journal of Nuclear Medicine*.

[81] http://www.fda.gov/medwatch/safety/2005/safety05.htm#NeutroSpec.

[82] Suntharalingam G., Perry M. R., Ward S. (2006). Cytokine storm in a phase 1 trial of the anti-CD28 monoclonal antibody TGN1412. *The New England Journal of Medicine*.

[83] Drazen J. M. (2006). Volunteers at risk. *The New England Journal of Medicine*.

[84] Zinn K. R., Chaudhuri T. R., Smyth C. A. (1999). Specific targeting of activated endothelium in rat adjuvant arthritis with a ^99m^Tc-radiolabeled E-selectin-binding peptide. *Arthritis & Rheumatism*.

[85] Gratz S., Béhé M., Boerman O. C. (2001). ^99m^Tc-E-selectin binding peptide for imaging acute osteomyelitis in a novel rat model. *Nuclear Medicine Communications*.

[86] Nößner E., Goldberg J. E., Naftzger C., Lyu S.-C., Clayberger C., Krensky A. M. (1996). HLA-derived peptides which inhibit T cell function bind to members of the heat-shock protein 70 family. *Journal of Experimental Medicine*.

[87] Broisat A., Riou L. M., Ardisson V. (2007). Molecular imaging of vascular cell adhesion molecule-1 expression in experimental atherosclerotic plaques with radiolabelled B2702-p. *European Journal of Nuclear Medicine and Molecular Imaging*.

[88] Nahrendorf M., Keliher E., Panizzi P. (2009). 18F-4V for PET-CT imaging of VCAM-1 expression in atherosclerosis. *JACC Cardiovascular Imaging*.

[89] Hatori A., Yui J., Yamasaki T. (2012). PET imaging of lung inflammation with [^18^F]FEDAC, a radioligand for translocator protein (18 kDa). *PLoS ONE*.

[90] Gaemperli O., Shalhoub J., Owen D. R. J. (2012). Imaging intraplaque inflammation in carotid atherosclerosis with ^11^C-PK11195 positron emission tomography/computed tomography. *European Heart Journal*.

[91] Chauveau F., Boutin H., van Camp N., Dollé F., Tavitian B. (2008). Nuclear imaging of neuroinflammation: a comprehensive review of [^11^C]PK11195 challengers. *European Journal of Nuclear Medicine and Molecular Imaging*.

[92] Gent Y. Y., Voskuyl A. E., Kloet R. W. (2012). Macrophage positron emission tomography imaging as a biomarker for preclinical rheumatoid arthritis: findings of a prospective pilot study. *Arthritis and Rheumatism*.

[93] van der Laken C. J., Elzinga E. H., Kropholler M. A. (2008). Noninvasive imaging of macrophages in rheumatoid synovitis using ^11^C-(*R*)-PK11195 and positron emission tomography. *Arthritis & Rheumatism*.

[94] Pottier G., Bernards N., Dollé F., Boisgard R. (2014). [^18^F]DPA-714 as a biomarker for positron emission tomography imaging of rheumatoid arthritis in an animal model. *Arthritis Research & Therapy*.

[95] Bernards N., Pottier G., Thézé B., Dollé F., Boisgard R. (2014). In vivo evaluation of inflammatory bowel disease with the aid of *μ*PET and the translocator protein 18 kDa radioligand [^18^F]DPA-714. *Molecular Imaging and Biology*.

[96] Immordino M. L., Dosio F., Cattel L. (2006). Stealth liposomes: review of the basic science, rationale, and clinical applications, existing and potential. *International Journal of Nanomedicine*.

[97] Torchilin V. P. (2005). Recent advances with liposomes as pharmaceutical carriers. *Nature Reviews Drug Discovery*.

[98] Dams E. T. M., Reijnen M. M. P. J., Oyen W. J. G. (1999). Imaging experimental intraabdominal abscesses with ^99m^Tc-PEG liposomes and ^99m^Tc-HYNIC IgG. *Annals of Surgery*.

[99] Laverman P., Dams E. T. M., Oyen W. J. G. (1999). A novel method to label liposomes with ^99m^Tc by the hydrazino nicotinyl derivative. *Journal of Nuclear Medicine*.

[100] Brouwers A. H., De Jong D. J., Dams E. T. M. (2000). Tc-99m-PEG-liposomes for the evaluation of colitis in Crohn's disease. *Journal of Drug Targeting*.

[101] Product Information Sheet for ABELCET 5 mg/mL Concentrate for Suspension for Infusion.

[102] (2012). *Product Information Sheet for AmBisome (amphotericin B) Liposome for Injection*.

[103] EMA/CHMP/806058/2009/Rev. 02 (2013). *Reflection Paper on the Data Requirements for Intravenous Liposomal Products Developed with Reference to an Innovator Liposomal Product*.

[104] FDA (2002). *Guidance for Industry, Liposome Drug Products, Chemistry, Manufacturing & Controls, Human Pharmacokinetics & Bioavailability, Labelling Documentation*.

[105] Lee J.-H., Cheng K. T., Malinin V. (2013). ^99m^Tc-labeled therapeutic inhaled amikacin loaded liposomes. *Journal of Liposome Research*.

[106] Underwood C., van Eps A. W., Ross M. W. (2012). Intravenous technetium-99m labelled PEG-liposomes in horses: a safety and biodistribution study. *Equine Veterinary Journal*.

[107] van de Veerdonk F. L., Netea M. G. (2013). New insights in the immunobiology of IL-1 family members. *Frontiers in Immunology*.

[108] van der Laken C. J., Boerman O. C., Oyen W. J. G. (1995). Specific targeting of infectious foci with radioiodinated human recombinant interleukin-1 in an experimental model. *European Journal of Nuclear Medicine*.

[109] Dinarello C. A., Simon A., Van Der Meer J. W. M. (2012). Treating inflammation by blocking interleukin-1 in a broad spectrum of diseases. *Nature Reviews Drug Discovery*.

[110] van der Laken C. J., Boerman O. C., Oyen W. J. G., van de Ven M. T. P., van der Meer J. W. M., Corstens F. H. M. (1998). Imaging of infection in rabbits with radioiodinated interleukin-1 (*α* and *β*), its receptor antagonist and a chemotactic peptide: a comparative study. *European Journal of Nuclear Medicine*.

[111] Barrera P., Van Der Laken C. J., Boerman O. C. (2000). Radiolabelled interleukin-1 receptor antagonist for detection of synovitis in patients with rheumatoid arthritis. *Rheumatology*.

[112] Prenant C., Cawthorne C., Fairclough M., Rothwell N., Boutin H. (2010). Radiolabeling with fluorine-18 of a protein, interleukin-1 receptor antagonist. *Applied Radiation and Isotopes*.

[113] Cawthorne C., Prenant C., Smigova A. (2011). Biodistribution, pharmacokinetics and metabolism of interleukin-1 receptor antagonist (IL-1RA) using [^18^F]-IL1RA and PET imaging in rats. *British Journal of Pharmacology*.

[114] Liu Z., wyffels L., Barber C., Hui M. M., Woolfenden J. M. (2011). A ^99m^Tc-labeled dual-domain cytokine ligand for imaging of inflammation. *Nuclear Medicine and Biology*.

[115] Geng X., Zhang R., Yang G., Jiang W., Xu C. (2012). Interleukin-2 and autoimmune disease occurrence and therapy. *European Review for Medical and Pharmacological Sciences*.

[116] Malek T. R., Castro I. (2010). Interleukin-2 receptor signalling: at the interface between tolerance and immunity. *Immunity*.

[117] Karczmarczyk U., Garnuszek P., Maurin M. (2010). Investigation of ^99m^Tc-labelling of recombinant human interleukin-2 via hydrazinonicotinamide. *Nuclear Medicine and Biology*.

[118] D'Alessandria C., di Gialleonardo V., Chianelli M. (2010). Synthesis and optimization of the labeling procedure of ^99m^Tc-Hynic-interleukin-2 for *in vivo* imaging of activated T lymphocytes. *Molecular Imaging and Biology*.

[119] di Gialleonardo V., Signore A., Glaudemans A. W. J. M., Dierckx R. A. J. O., de Vries E. F. J. (2012). *N*-(4-^18^F-fluorobenzoyl)interleukin-2 for PET of human-activated T lymphocytes. *Journal of Nuclear Medicine*.

[120] Opalinska M., Stompor T., Pach D. (2012). Imaging of inflamed carotid artery atherosclerotic plaques with the use of ^99m^Tc-HYNIC-IL-2 scintigraphy in end-stage renal disease patients. *European Journal of Nuclear Medicine and Molecular Imaging*.

[121] Signore A., Chianelli M., Annovazzi A. (2000). ^123^I-Interleukin-2 scintigraphy for in vivo assessment of intestinal mononuclear cell infiltration in Crohn's disease. *Journal of Nuclear Medicine*.

[122] Campbell L. M., Maxwell P. J., Waugh D. J. J. (2013). Rationale and means to target pro-inflammatory interleukin-8 (CXCL8) signaling in cancer. *Pharmaceuticals*.

[123] Moser B. (2003). Chemokines: role in immune cell traffic. *European Cytokine Network*.

[124] Rennen H. J. J. M., Boernan O. C., Oyen W. J. G., Van der Meer J. W. M., Corstens F. H. M. (2001). Specific and rapid scintigraphic detection of infection with ^99m^Tc-labeled interleukin-8. *Journal of Nuclear Medicine*.

[125] Gratz S., Rennen H. J. J. M., Boerman O. C., Oyen W. J. G., Corstens F. H. M. (2001). Rapid imaging of experimental colitis with ^99m^Tc-interleukin-8 in rabbits. *Journal of Nuclear Medicine*.

[126] Gratz S., Rennen H. J. J. M., Boerman O. C., Oyen W. J. G., Burma P., Corstens F. H. M. (2001). ^99m^Tc-interleukin-8 for imaging acute osteomyelitis. *Journal of Nuclear Medicine*.

[127] Rennen H. J. J. M., Bleeker-Rovers C. P., Van Erd J. E. M. (2004). ^99m^Tc-labeled interleukin-8 for scintigraphic detection of pulmonary infections. *Chest*.

[128] Bleeker-Rovers C. P., Rennen H. J. J. M., Boerman O. C. (2007). 99mTc-labeled interleukin 8 for the scintigraphic detection of infection and inflammation: first clinical evaluation. *Journal of Nuclear Medicine*.

[129] Rennen H. J. J. M., Frielink C., Brandt E. (2004). Relationship between neutrophil-binding affinity and suitability for infection imaging: comparison of ^99m^Tc-labeled NAP-2 (CXCL-7) and 3 C-terminally truncated isoforms. *Journal of Nuclear Medicine*.

[130] Barrie A. M., Plevy S. E. (2005). The interleukin-12 family of cytokines: therapeutic targets for inflammatory disease mediation. *Clinical and Applied Immunology Reviews*.

[131] Hamza T., Barnett J. B., Li B. (2010). Interleukin 12 a key immunoregulatory cytokine in infection applications. *International Journal of Molecular Sciences*.

[132] Annovazzi A., D'Alessandria C., Bonanno E. (2006). Synthesis of ^99m^Tc-HYNIC-interleukin-12, a new specific radiopharmaceutical for imaging T lymphocytes. *European Journal of Nuclear Medicine and Molecular Imaging*.

[133] Monk P. N., Scola A.-M., Madala P., Fairlie D. P. (2007). Function, structure and therapeutic potential of complement C5a receptors. *British Journal of Pharmacology*.

[134] van Eerd J. E. M., Boerman O. C., Corstens F. H. M., Oyen W. J. G. (2003). Radiolabeled chemotactic cytokines: new agents for scintigraphic imaging of infection and inflammation. *Quarterly Journal of Nuclear Medicine*.

[135] Huey R., Hugli T. E. (1985). Characterization of a C5a receptor on human polymorphonuclear leukocytes (PMN). *Journal of Immunology*.

[136] Rennen H. J., Oyen W. J., Cain S. A., Monk P. N., Corstens F. H., Boerman O. C. (2003). Tc-99m-labeled C5a and C5a des Arg74 for infection imaging. *Nuclear Medicine and Biology*.

[137] Welling M. M., Nibbering P. H., Paulusma-Annema A., Hiemstra P. S., Pauwels E. K. J., Calame W. (1999). Imaging of bacterial infections with ^99m^Tc-labeled human neutrophil peptide-1. *Journal of Nuclear Medicine*.

[138] Welling M. M., Paulusma-Annema A., Balter H. S., Pauwels E. K. J., Nibbering P. H. (2000). Technetium-99m labelled antimicrobial peptides discriminate between bacterial infections and sterile inflammations. *European Journal of Nuclear Medicine*.

[139] Welling M. M., Lupetti A., Balter H. S. (2001). ^99m^Tc-labeled antimicrobial peptides for detection of bacterial and *Candida albicans* infections. *Journal of Nuclear Medicine*.

[140] Ferro-Flores G., Ramírez F. D. M., Meléndez-Alafort L., Murphy C. A. D., Pedraza-López M. (2004). Molecular recognition and stability of ^99m^Tc-UBI 29-41 based on experimental and semiempirical results. *Applied Radiation and Isotopes*.

[141] Welling M. M., Korsak A., Gorska B. (2005). Kit with technetium-99m labelled antimicrobial peptide UBI 29–41 for specific infection detection. *Journal of Labelled Compounds and Radiopharmaceuticals*.

[142] Akhtar M. S., Qaisar A., Irfanullah J. (2005). Antimicrobial peptide ^99m^Tc-Ubiquicidin 29-41 as human infection-imaging agent: Clinical trial. *Journal of Nuclear Medicine*.

[143] Huub J. J. M., Rennen O. C., Boerman W. J. G., Oyen W. J., Corstens F. H. M. (2002). Scintigraphic imaging of inflammatory processes. *Current Medicinal Chemistry-Anti-Inflammatory & Anti-Allergy Agents*.

[144] Babich J. W., Tompkins R. G., Graham W., Barrow S. A., Fischman A. J. (1997). Localization of radiolabeled chemotactic peptide at focal sites of *Escherichia coli* infection in rabbits: evidence for a receptor-specific mechanism. *Journal of Nuclear Medicine*.

[145] Fischman A. J., Rauh D., Solomon H. (1993). In vivo bioactivity and biodistribution of chemotactic peptide analogs in nonhuman primates. *Journal of Nuclear Medicine*.

[146] Lupetti A., Pauwels E. K. J., Nibbering P. H., Welling M. M. (2003). ^99m^Tc-Antimicrobial peptides: Promising candidates for infection imaging. *Quarterly Journal of Nuclear Medicine*.

[147] http://www.imgt.org/IMGTrepertoire/GenesClinical/monoclonalantibodies/.

